# Palaeoecological differences underlie rare co-occurrence of Miocene European primates

**DOI:** 10.1186/s12915-020-00939-5

**Published:** 2021-01-19

**Authors:** Daniel DeMiguel, Laura Domingo, Israel M. Sánchez, Isaac Casanovas-Vilar, Josep M. Robles, David M. Alba

**Affiliations:** 1 ARAID foundation / Universidad de Zaragoza, Departamento de Ciencias de la Tierra, and Instituto Universitario de Investigación en Ciencias Ambientales de Aragón (IUCA), Pedro Cerbuna 12, 50009 Zaragoza, Spain; 2 Institut Català de Paleontologia Miquel Crusafont, Universitat Autònoma de Barcelona, Edifici ICTA-ICP, C/ Columnes s/n, Campus de la UAB, 08193 Cerdanyola del Vallès, Barcelona, Spain; 3 Departamento de Geodinámica, Estratigrafía y Paleontología Facultad de Ciencias Geológicas, Universidad Complutense de Madrid, José Antonio Novais 12, 28040 Madrid, Spain; 4 Earth and Planetary Sciences Department, University of California Santa Cruz, 1156 Hight Street, Santa Cruz, CA 95064 USA

**Keywords:** Hominoids, Pliopithecoids, Primate evolution/adaptation, Palaeodiet, Stable isotopes, Tooth wear, Feeding behaviour, Palaeobiology

## Abstract

**Background:**

The two main primate groups recorded throughout the European Miocene, hominoids and pliopithecoids, seldom co-occur. Due to both their rarity and insufficiently understood palaeoecology, it is currently unclear whether the infrequent co-occurrence of these groups is due to sampling bias or reflects different ecological preferences. Here we rely on the densely sampled primate-bearing sequence of Abocador de Can Mata (ACM) in Spain to test whether turnovers in primate assemblages are correlated with palaeoenvironmental changes. We reconstruct dietary evolution through time (ca. 12.6–11.4 Ma), and hence climate and habitat, using tooth-wear patterns and carbon and oxygen isotope compositions of enamel of the ubiquitous musk-deer *Micromeryx*.

**Results:**

Our results reveal that primate species composition is strongly correlated with distinct environmental phases. Large-bodied hominoids (dryopithecines) are recorded in humid, densely-forested environments on the lowermost portion of the ACM sequence. In contrast, pliopithecoids inhabited less humid, patchy ecosystems, being replaced by dryopithecines and the small-bodied *Pliobates* toward the top of the series in gallery forests embedded in mosaic environments.

**Conclusions:**

These results support the view that pliopithecoid primates preferred less humid habitats than hominoids, and reveal that differences in behavioural ecology were the main factor underpinning their rare co-occurrence during the European Miocene. Our findings further support that ACM hominoids, like Miocene apes as a whole, inhabited more seasonal environments than extant apes. Finally, this study highlights the importance of high-resolution, local investigations to complement larger-scale analyses and illustrates that continuous and densely sampled fossiliferous sequences are essential for deciphering the complex interplay between biotic and abiotic factors that shaped past diversity.

## Background

Fossil primates from the Miocene of Europe are generally rare and absent from most sites, and when recorded, different primate species only seldom co-occur within a single locality (stratigraphic horizon). As a result, there is an ongoing debate about the factors underpinning the geographic and chronostratigraphic distribution of Miocene primates in this continent [1–6]. Before the dispersal of cercopithecoids (Old World monkeys) into Europe by the early Turolian (ca. 8.5 Ma, late Miocene), two main groups are recorded there: pliopithecoids, generally considered a Eurasian clade of stem catarrhines (i.e. preceding the cercopithecoid-hominoid split [7]), and hominoids (crown catarrhines more closely related to extant apes and humans than to cercopithecoids [8–10]). Both groups presumably dispersed from Africa to Eurasia following the closure of the Tethys Seaway during the late middle Miocene and subsequently diversified across the continent giving rise to multiple genera and species. There are approximately one hundred known localities recording either or both of these groups—almost 20% corresponding to Abocador de Can Mata (ACM) in Spain—although they only co-occur in less than 10% of them, with pliopithecoid-bearing localities being slightly more abundant (ca. 55 vs. 45%) than hominoid-bearing ones [4, 5]. European hominoids are generally larger than pliopithecoids and considered great apes (hominids), except for the small-bodied *Pliobates*, interpreted as a stem hominoid [11].

There are very few sites in the European Miocene where hominoids and pliopithecoids co-occur [1, 4, 5], and in most cases, fossils of each group come from different localities within the same site (e.g. different karstic fissure fillings from La Grive) or it is uncertain whether their remains came from the same stratigraphic horizon (e.g. Castell de Barberà [6]). Strong taphonomic evidence supporting sympatry is only available from Rudabánya in Hungary [12] and ACM (locality ACM/C5-C3 [5]). These localities therefore offer a unique opportunity to evaluate the palaeoenvironmental conditions that enabled the coexistence of pliopithecoids and hominoids. Given the rarity of primate remains among mammalian assemblages from the European Miocene, the infrequent co-occurrence of two different primate species at a single locality might be, at least in part, a sampling artefact [1]. However, the lack of co-occurrence in many well-sampled primate-bearing localities would rather support the view that their infrequent coexistence is a real phenomenon that requires an explanation.

The competitive exclusion principle [13] predicts that species occupying the same ecological niche cannot coexist on the long-term, ultimately leading to the prevalence of one over the other, or to the progressive divergence of their respective niches. This explanation is unlikely to hold for different clades such as pliopithecoids and hominoids, characterised among others by different locomotor adaptations—leading to the proposal that these two groups probably had different habitat preferences, which only enabled their coexistence under particular ecological conditions [1]. Early ecomorphological analyses based on ungulate hypsodonty (a proxy for vegetation structure also used to infer palaeoprecipitation) have concluded that both hominoid and pliopithecoid-bearing localities from the European Miocene were more humid than those lacking primates [2]. More recent work based on hypsodonty further showed that pliopithecoids generally inhabited more humid environments (i.e. with higher moisture and/or rainfall) than hominoids, although probably less humid than those in which both groups co-occur [4]. However, given the small number of fossil localities recording both taxa, such comparisons lack statistical power and may fail to consider palaeoenvironmental differences across geography and time throughout the Miocene, especially at the regional and local scales.

**Fig. 1:**
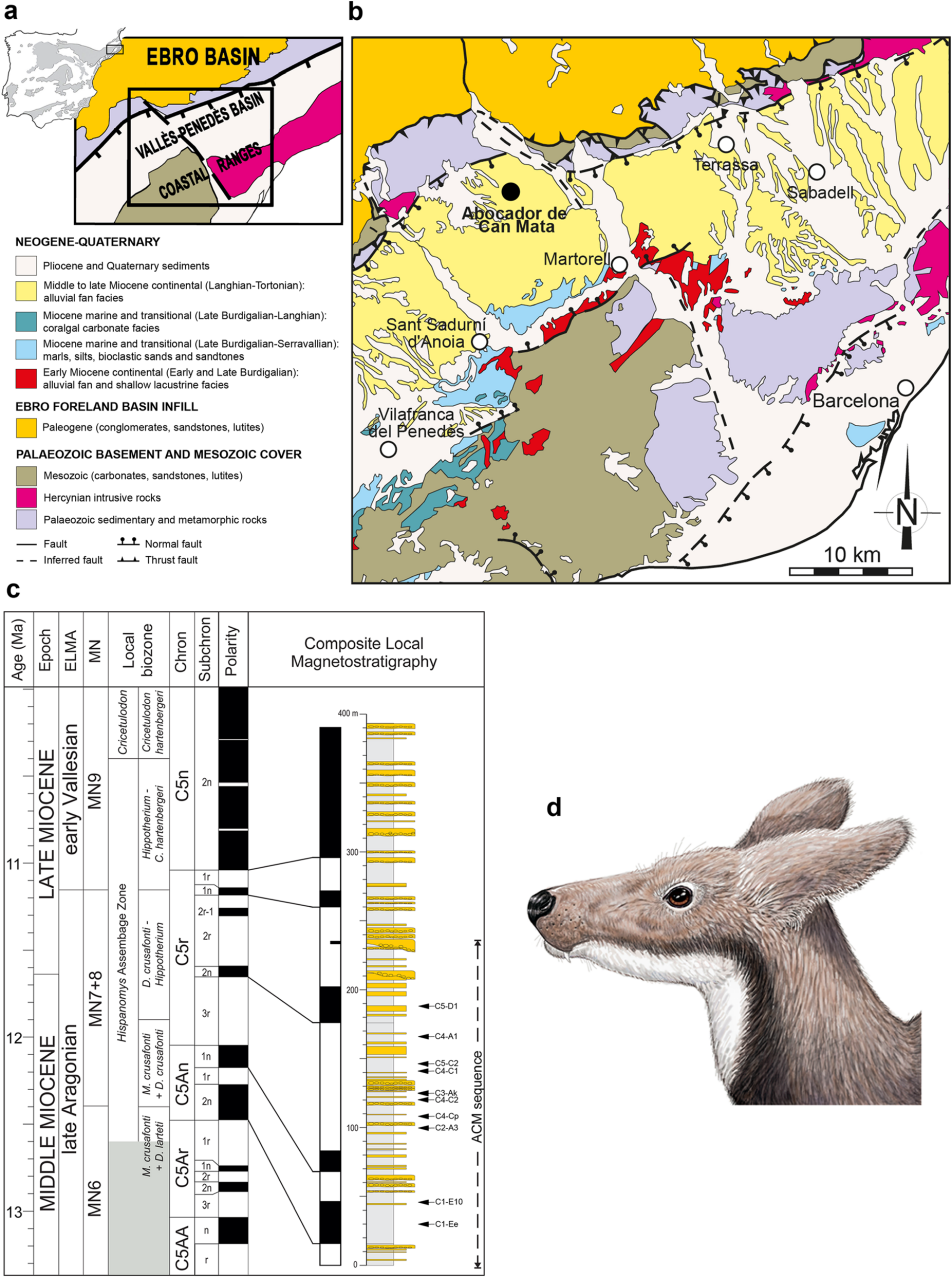
Abocador de Can Mata (ACM) and the moschid *Micromeryx*. **a** Geographical situation and general geological context of the Vallès-Penedès Basin. **b** Detailed geological map of the basin and the sequence of Abocador de Can Mata (ACM) (black dot). **c** Correlation of the composite local magnetostratigraphy of ACM series with the Geomagnetic Polarity Time Scale (modified from Alba et al. [5]). European Land Mammal Ages, Mammal Neogene (MN) units and local biozones of the Vallès-Penedès Basin are shown on the left. The shadowed region indicates an unsampled interval of the Vallès-Penedès record. The stratigraphic positions of the ACM localities studied in this work are shown to the right on the composite lithostratigraphic column. Note that the bottom boundary of the lowermost local biozone is unknown. **d** Life reconstruction of a *Micromeryx azanzae* male. Art by I.M.S

Focusing on faunal elements that accompany primates within a single area and over a restricted time span would allow us to test whether turnovers in the primate assemblage are correlated to local changes in palaeoenvironmental conditions. The composite stratigraphic sequence of ACM, located in the area of els Hostalets de Pierola within the Vallès-Penedès Basin (NE Iberian Peninsula [14]) (Fig. 1a–c), and spanning more than 1 Myr (12.6–11.4 Ma [5, 15]), offers an unparalleled opportunity to test this hypothesis for several reasons. First, the ACM sequence has delivered one of the most diverse primate assemblages from the European Miocene, including both hominoids and pliopithecoids [5]. Second, the co-occurrence of hominoids and pliopithecoids has only been recorded in one out of the 19 ACM primate-bearing localities, and the distribution of each group throughout the series does not appear random [5]. Finally, thanks to continuous palaeontological surveillance during the construction of a landfill, most of the fossil finds are accurately dated based on detailed litho-, bio- and magnetostratigraphic correlations [5, 16, 17]. This offers the opportunity to test alternative explanations for the variable temporal distribution of both primate groups during a restricted time span and within a uniform depositional setting.

With this aim in mind, here we present a reconstruction of the local climate and palaeoenvironments through the ACM sequence based on tooth wear and dental enamel stable carbon and oxygen isotope values (represented by the notation δ^13^C and δ^18^O) of the ruminant *Micromeryx*—(Fig. 1d and Figure S1)—a representative of the family Moschidae (musk-deer) [18, 19]. Because the diet of any plant-eating mammal is a direct link with the habitats in which it lives, we used the diet (i.e. ecology) of this ruminant to inform about ACM primate ecological preferences and habitats. The selection of *Micromeryx* is based on the following reasons: (1) the record of this taxon throughout the ACM stratigraphic sequence, characterised by abundant isolated teeth and dentognathic fragments, allows us to construct a continuous tooth-wear and isotopic record; (2) by focusing on a single genus, we can characterise more consistently changes in the vegetation cover, food abrasiveness, etc., across the selected time interval, avoiding thus biases due to different physiologies; and (3) *Micromeryx* was ubiquitous in the Miocene of Iberia, inhabiting a varied range of biomes from more or less open savannas to (sub) tropical forests [18, 20], and exhibiting an extraordinary versatility in terms of exploitation of nutrients and resources. The combination of all these factors justifies the suitability of employing *Micromeryx* as a case study to investigate the environmental and climatic shifts that took place during the latest middle Miocene in the area of els Hostalets de Pierola.

## Results

### Tooth wear

The fossil material studied consists of dentognathic remains and isolated teeth of *Micromeryx*. Although initially a single species of *Micromeryx* was reported from ACM [15], the currently available dental material indicates the presence of three different morphotypes that likely represent different species (Additional file 1: Supplementary information, Note 1).

For mesowear, we measured individuals and provide the results for the three morphotypes separately (Additional file 1: Supplementary information, Note 2). All *Micromeryx* morphotypes (Table 1) show occlusal surfaces with predominance of high relief (pH = 95–100%) and sharpened cusps (pS = 69–87%), although there is a considerable proportion of rounded apices (pR = 13–31%). Morphotypes do not have any incidence of blunt cusps or, except for *Micromeryx* morphotype 3 (pL = 5%), low occlusal relief (which relates to a low height difference between tooth cusps and valleys). Average mesowear score (MS) for morphotypes ranges from 0.19 to 0.31 (Table 1). We do find significant differences with the chi-square test (*χ*^2^) but marginally non-significant with the Fisher exact test. For the chi-square test, the results show that *Micromeryx* morphotype 1 is different from morphotype 2 (*p* = 0.0283), whereas non-significant differences are between morphotypes 2 and 3 (*p* = 0.2255) and between morphotypes 1 and 3 (*p* = 0.2019). On average, mesowear results indicate a browsing on soft vegetation and low levels of abrasives (endogenous phytolith-rich grasses and dicotyledonous, and exogenous dust and grit), although *Micromeryx* morphotype 1—with more rounded cusps and higher MS (Table 1)—shows a shift toward the exploitation of tougher and more abrasive foods than the others.

**Table 1:** Summary of mesowear and isotopic values of *Micromeryx* from the ACM sequence according to morphotypes and environmental phases

**Morphotypes**	# **M**	**pS**	**pR**	**pH**	**MS**	#**C**	**δ**^**13**^**C**	**SD δ**^**13**^**C**	**δ**^**18**^**O**_**CO3**_	**SD δ**^**18**^**O**_**CO3**_	#**P**		**δ**^**18**^**O**_**PO4**_		**SD δ**^**18**^**O**_**PO4**_	**Δ δ**^**18**^**O**_**CO3**_**-δ**^**18**^**O**_**PO4**_		
**Morphotype 1**	8	69.2	30.8	100	0.31	8	− 10.5	0.7	27.9	1.0	6		19.6		1.4	8.4		
**Morphotype 2**	24	80.6	19.4	100	0.19	21	− 11.8	0.8	27.7	1.5	19		19.1		1.9	8.7		
**Morphotype 3**	11	86.7	13.3	95	0.21	6	− 11.0	1.2	27.0	0.6	5		18.5		1.1	8.3		
**Environmental phases**	#**M**	**pS**	**pR**	**pH**	**MS**	#**C**	**δ**^**13**^**C**	**SD δ**^**13**^**C**	**δ**^**18**^**O**_**CO3**_	**SD δ**^**18**^**O**_**CO3**_	#**P**	**δ**^**18**^**O**_**PO4**_	**SD δ**^**18**^**O**_**PO4**_	**Δ δ**^**18**^**O**_**CO3**_**-δ**^**18**^**O**_**PO4**_	**δ**^**13**^**C**_**diet, mequ**_	**MAP**^**a**^ **(mm/year)**	**MAP**^**b**^ **(mm/year)**	**MAT (**°**C)**
**Phase III (11.70–11.60 Ma)**	23	71.9	28.1	97.3	0.32	19	− 11.1	1.2	27.2	1.2	19	18.5	1.6	8.7	− 27.2	801	608	17.1
**Phase II (11.90–11.79 Ma)**	15	83.3	16.7	100	0.16	13	− 11.4	1.2	27.7	1.2	6	19.8	1.7	8.2	− 27.5	992	765	20.3
**Phase I (12.33–11.95 Ma)**	7	90.9	9.1	100	0.09	5	− 12.0	0.3	28.9	0.8	5	20.4	0.9	8.5	− 28.1	1190	928	21.8

#M (number of samples for mesowear); percentage of specimens with sharp (pS) and rounded (pR) cusps; percentage of specimens with high (pH) occlusal relief; mesowear score (MS); #C (number of samples for stable isotope analyses on the carbonate fraction); mean δ^13^C (‰ VPDB); standard deviation (SD) δ^13^C (‰ VPDB); mean δ^18^O_CO3_ (‰ VSMOW); standard deviation (SD) δ^18^O_CO3_ (‰ VSMOW); #P (number of samples for stable isotope analyses on the phosphate fraction); mean δ^18^O_PO4_ (‰ VSMOW); standard deviation (SD) δ^18^O_PO4_ (‰ VSMOW); Δδ^18^O_CO3_ − δ^18^O_PO4_, mean δ^13^C_diet, mequ_ (‰ VPDB); inferred mean MAP (mm/year) (from Kohn [21]) without (estimated MAP^a^) and with (estimated MAP^b^) altitude and latitude correction; and inferred mean MAT (°C)

### Stable isotope data

The difference between carbonate (δ^18^O_CO3_) and phosphate (δ^18^O_PO4_) oxygen isotopic composition can be used to monitor possible bioapatite diagenetic alteration. *Micromeryx* tooth enamel did not undergo extensive post-burial alteration since the difference calculated between δ^18^O_CO3_ and δ^18^O_PO4_ (∆^18^O_CO3_-_PO4_ = δ^18^O_CO3_ − δ^18^O_PO4_) values for the whole dataset (8.6 ± 0.8‰) is within the range obtained when considering modern mammals (~ 8.6–9.1‰ [22, 23]) (Additional file 1: Supplementary information, Note 3).

*Micromeryx* morphotypes (Table 1 and Additional file 6: Table S1) yielded tooth enamel δ^13^C values indicative of woodland to woodland-mesic C_3_ grassland conditions (see Additional file 1: Supplementary information, Note 4 for a detailed explanation of the calculated δ^13^C cut-off values among different habitats). Significant differences in δ^13^C values have been only found between morphotypes 1 and 2 of *Micromeryx* (*t* = 4.250, *p* < 0.001) (Additional file 7: Table S2). Tooth enamel δ^18^O_CO3_ and δ^18^O_PO4_ values do not show significant differences among the three morphotypes (δ^18^O_CO3_: *F* = 0.845, df = 2, *p* = 0.439, and δ^18^O_PO4_: *F* = 0. 562, df = 2, *p* = 0.577) (Additional file 7: Table S2).

### Relationship between mesowear and δ^13^C values

**Fig. 2:**
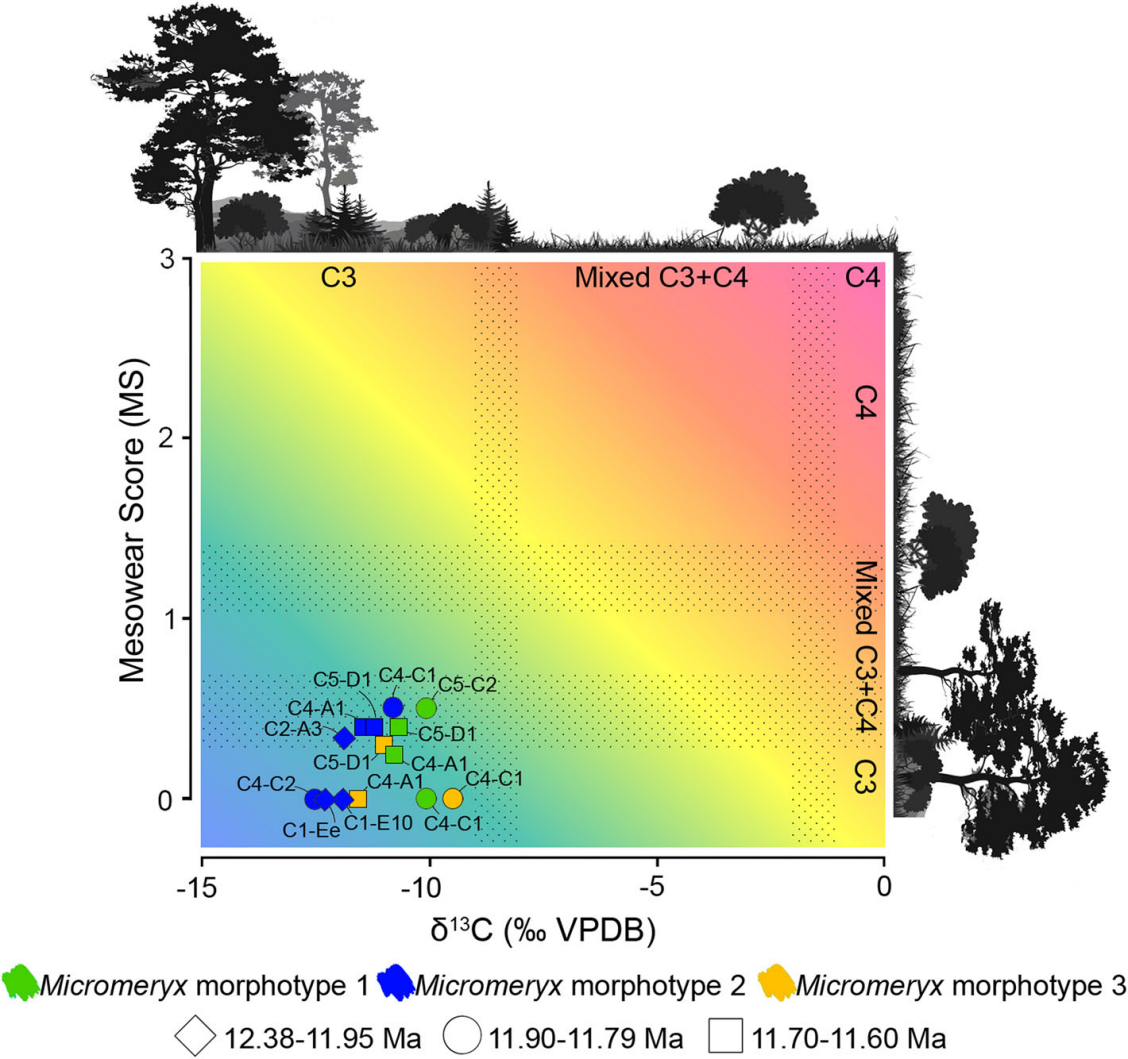
Scatter plot of mesowear and δ^13^C values (‰ VPDB) of *Micromeryx.* Stippled areas show the transition between C_3_-dominated diets, mixed C_3_-C_4_ diets and C_4_-dominated diets. Colour informs about *Micromeryx* morphotype (green for *Micromeryx* morphotype 1, blue for *Micromeryx* morphotype 2 and yellow for *Micromeryx* morphotype 3), and symbol refers to the temporal range within the sedimentary sequence (diamond for 12.38–11.95 Ma, circle for 11.90–11.79 Ma and square for 11.70–11.60 Ma). See Additional file 1: Supplementary information, Notes 2 and 3 for further details

A scatter plot showing the correlation between mean MS and mean δ^13^C (‰ VPDB) among *Micromeryx* morphotypes by localities was constructed (Fig. 2). Niche domains are visually presented for each variable. This approach (described in Additional file 1: Supplementary information, Notes 2 and 4) allowed us to contextualise the niche occupation per morphotype and locality given the variables investigated. MS and δ^13^C values point to a frequent ingestion of C_3_ plants in woodland to mesic C_3_ grasslands. Mean MS of some individuals of morphotypes 1 and 2 from a few localities (those with MS = 0.33 to 0.5) is also compatible with regular consumption of C_4_ vegetation. C_4_ plants have never been documented as an important component of plant communities in the Iberian Neogene (despite being recorded there since the Oligocene) [24, 25]. However, they may well have been present to some extent in some areas and/or time intervals (e.g. ACM/C4-C1 and ACM/C5-C2 at ~ 11.8 Ma).

## Discussion

### *Micromeryx* diet at ACM

Our results indicate that the bulk of *Micromeryx* diet at ACM consisted of foliage with a particular emphasis on forbs, dicots and woody leaves. The various morphotypes generally maintain sharpened, high-relief cusp apices and low MS—a signal that informs that foods were of relatively low abrasion, as seen in extant forest-dweller browsers [26]. We rule out a regular consumption of fruits and/or seeds in ACM, as teeth show no signs of strong rounding or blunt apices—and frugivorous taxa have significant percentages of rounded and blunt cusps because of tip-crushing [27]. These results contrast with some previous data for middle and late Miocene *Micromeryx* from elsewhere in Europe, which appear strictly frugivorous [28–30]. However, these results are in agreement with the leaf browsing inferred for other *Micromeryx* [29, 31]. Therefore, it seems that *Micromeryx*, since its oldest occurrences in the middle Miocene of Eurasia, was capable of feeding alternatively on fruits, seeds and soft leaves, depending on habitat-specific circumstances (e.g. ecologic niche partitioning or food availability). In the case of ACM, the unusual secondary crests of the upper molars of *Micromeryx* are compatible with an adaptation for heavier reliance on leaves and stems, as seen in other mammalian groups [32]—an anatomical trait that is consistent with the folivorous signal retrieved from mesowear analyses. The *Micromeryx* from ACM are therefore the only ones in which these features are recognised, probably showing a regional adaptation associated with the particularity of these environments. Moreover, *Micromeryx* had a wider dietary plasticity than modern *Moschus*, whose diet comprises mainly arboreal lichens (a resource rarely exploited by other ruminants), forbs and woody leaves [33].

Within such a generalised soft, leafy browsing, there are differences among morphotypes in tooth wear and isotopic values through time (Additional file 6: Table S1). In other words, the same morphotype behaves differently when the temporal gradient is considered. The less sharp and more rounded cusps of *Micromeryx* morphotypes 1 and 2 recorded from 11.81 Ma onwards (and a signal of browse-dominated mixed feeding for some individuals) reflect a more pronounced abrasion than in older specimens and indicate that more abrasive browse and/or some dust/grit-infested foliage was eaten—as extant browsers that feed on leafy, soft foods generally maintain sharpened/high relief cusps [27, 31] (Additional file 6: Table S1). That is, abrasive browse and encroachments by exogenous dust/grit content was only slight or even absent in the diet of older *Micromeryx*, whereas it significantly increased in later forms along the ACM sequence. δ^13^C data support dietary inferences based on mesowear, as all *Micromeryx* morphotypes depict values that are within woodland to woodland-mesic C_3_ grassland conditions, thereby indicating a consumption of both soft leaves and more abrasive grasses.

### Temporal patterns in *Micromeryx* tooth wear and stable isotopes along the ACM sequence

**Fig. 3:**
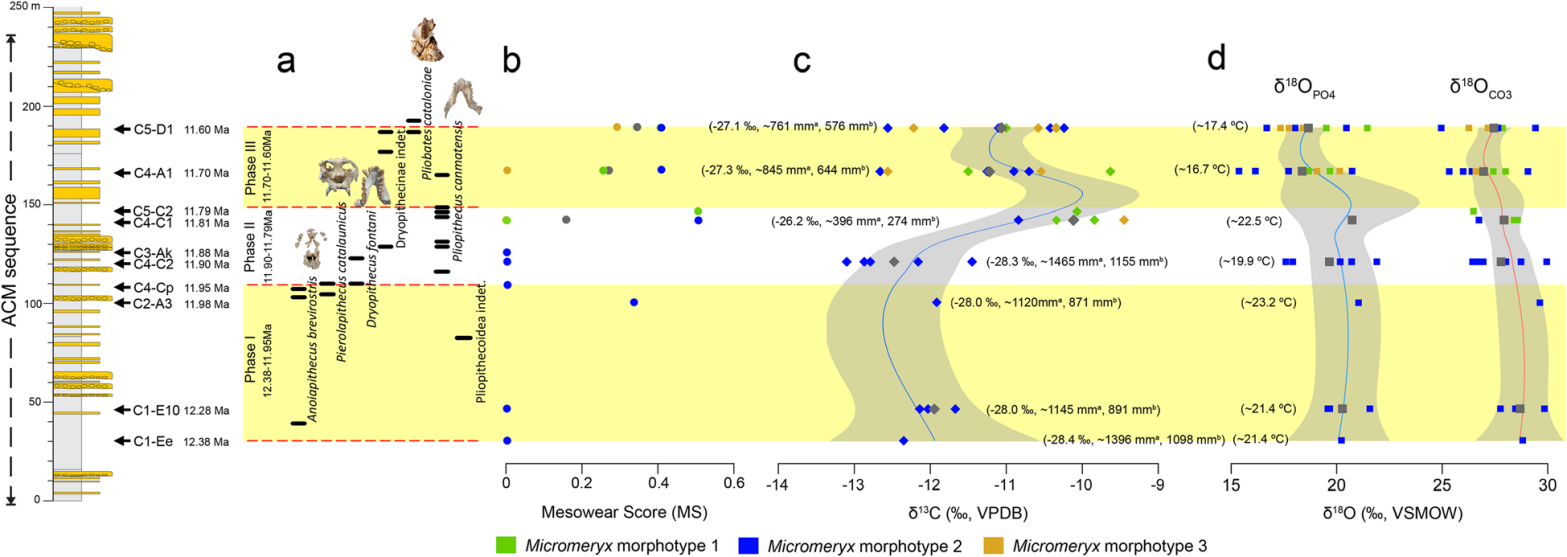
Correlation of mesowear and isotopic values of *Micromeryx* arranged temporally along the ACM sequence. **a** Environmental phases, separated by dashed lines, and stratigraphic ranges of the primates recovered at ACM based on occurrence in localities. **b** Average mesowear scores (MS) by locality for *Micromeryx* morphotypes. **c** *Micromeryx* tooth enamel raw and mean δ^13^C (‰ VPDB). Calculated average modern equivalent of diet composition (δ^13^C_diet, meq_, ‰ VPDB) and estimated MAP (mm/year) values (without [MAP^a^] and with [MAP^b^] altitude and latitude correction) are given in parentheses. **d** *Micromeryx* tooth enamel raw and mean δ^18^O_CO3_ and δ^18^O_PO4_ (‰ VSMOW) values. Calculated MAT (°C) values are given in parentheses. Locally weighted polynomial lines are fitted to isotopic data. Calculations further include the 95% confidence region. Colour symbols are for raw data and grey symbols are for mean values. See Additional file 1: Supplementary information, Note 4 for further details

The primate assemblage recorded at ACM [5] includes three great ape (dryopithecine) species from different genera (*Pierolapithecus catalaunicus*, *Anoiapithecus brevirostris* and *Dryopithecus fontani*) [5, 10, 16, 34], the pliopithecoid *Pliopithecus canmatensis* [35], probably a second pliopithecoid unassigned to species [36] and a putative stem hominoid (*Pliobates cataloniae*) [11], alternatively interpreted as a pliopithecoid [37]. The distribution of these taxa is not homogeneous along the ACM sequence, with *Pliopithecus* postdating most great ape finds but preceding *Pliobates* [5] (Fig. 3a).

We show mesowear and isotopic data of *Micromeryx* teeth in chronological order of the localities for a time span ranging from 12.38 to 11.63 Ma (Fig. 3), to compare their chronostratigraphic distribution with palaeoenvironmental changes through time. While remaining within a browsing dietary category based on C_3_ plants, differences observed in both mesowear and isotopic values among *Micromeryx* specimens provide insight on the environmental changes along the ACM temporal sequence during the latest middle Miocene. This is particularly relevant given that the lack of other palaeoenvironmental proxies from this area (such as pollen or macroplant remains) hinders a more precise reconstruction of the vegetation structure and other characteristics of the various habitats occupied by primates.

Overall, the data reported here indicate that ACM habitats became progressively less humid and more heterogeneous (or, at least, are characterised by a gradient toward less dense canopy structure and more open patches), as reflected by (*i*) a trend toward higher values of mesowear—from the sharpest cusps and low MS of 0 at 12.38 Ma (ACM/C1-Ee) to more intermediate (more rounded) cusp morphologies and higher MS around 0.5 at 11.60 Ma (ACM/C5-D1) (Fig. 3 (b)); (*ii*) an increase in mean *Micromeryx* δ^13^C values (from − 12.4‰ in ACM/C1-Ee to − 11.1‰ in ACM/C5-D1; Fig. 3 (c)); and (*iii*) a high variability in the type of vegetation consumed—revealed by a wider range of δ^13^C values in the youngest localities and fuelled by the coexistence of the three morphotypes (see a change in standard deviation values in Table 1). The increase in mean δ^13^C values may have been driven by two phenomena: a shift toward drier habitats, including non-forest patches, or, alternatively, a greater reliance on fruits through time. A change toward a more frugivorous diet would have led to a slight increase in *Micromeryx* tooth enamel δ^13^C, as a significant consumption of fruits ultimately results in higher bioapatite δ^13^C values [38, 39]. There is, however, little reason to support strong frugivory for ACM *Micromeryx*, as this is contradicted by their attrition-dominated mesowear patterns. This does not mean that fruits were unavailable at ACM. In fact, all of the primate species recorded relied on frugivory to a large extent, even if with a different emphasis on hard-object feeding depending on the species [11, 40, 41]. However, a drop in the estimated mean annual precipitation (MAP) values throughout the sequence (from ~ 1395 to ~ 762 mm/year or from ~ 1097 to ~ 575 mm/year with altitude and latitude correction; Table 1, Fig. 3 (c), Additional file 6: Table S1) supports the fact that fruits were preferentially exploited by arboreal, or at least semiterrestrial, species instead of terrestrial taxa such as *Micromeryx*.

Although the oxygen isotope composition does not vary significantly through time, there is a slight decrease in both δ^18^O_CO3_ and δ^18^O_PO4_ values (from − 28.8‰ and 20.3‰ in ACM/C1-Ee to 27.4‰ and 18.7‰ in ACM/C5-D1; Fig. 3 (c, d)). Oxygen isotope composition of carbonate (δ^18^O_CO3_) and phosphate (δ^18^O_PO4_) fractions of tooth enamel reflects δ^18^O of body water (δ^18^O_bw_) [42, 43]. Changes associated with δ^18^O_bw_ value mirror variations in the isotopic composition of ingested water, either through drinking or plant water (in the case of herbivores). *Micromeryx* δ^18^O values are less likely to vary according to physiological factors like fractionated water loss through the lungs or skin, since this is a relatively mesic environment overall, and the species are closely related over a narrow time window. When considering extinct mammals such as *Micromeryx*, it is difficult to assess the type of water economy they may have had, due to the lack of modern analogues. The extant sister group of *Micromeryx* within the Moschidae is the genus *Moschus* (musk deer) [44], which inhabits forest and mountainous parts of Asia [33, 45] and has a browsing diet, although it also has the ability to cope with poorer, less nutritious foods when high-quality forage is in short supply, such as in winter [33, 46]. Independently from its dietary behaviour, *Moschus* has been observed to drink water on a daily basis (Prikhod’ko, pers. comm.); therefore, its tooth enamel δ^18^O signal will largely be dependent on drinking water δ^18^O values. On the assumption that the water reliance of *Moschus* is applicable to *Micromeryx*, mean annual temperature (MAT) values have been estimated based on *Micromeryx* tooth enamel δ^18^O_PO4_ values. They show a decreasing trend along the ACM sequence from 21.4 °C in ACM/C1-Ee to 17.4 °C in ACM/C5-D1 (Table 1, Fig. 3 (d), Additional file 6: Table S1). This trend toward lower temperatures may be framed within the gradual cooling that started by 14 Ma after the Mid-Miocene Climatic Optimum [47]. In the Iberian Peninsula, this long interval, which coincided with the expansion of mesothermic deciduous vegetation and the extinction or significant decrease in abundance of thermophilous evergreen plants [48, 49], witnessed an increase in the diversity of moschids [18].

### Primate assemblage composition in relation to palaeoenvironmental changes

Our analyses further show a fluctuation in diet composition for *Micromeryx* individuals, revealing the existence of three distinct environmental phases in ACM (Table 1, Fig. 3 (a–d)), with temporal patterns in precipitation, temperature and aridity that relate to changes in primate assemblage composition.

#### Phase I

A first phase ranges from the beginning of the sequence (12.38 Ma, ACM/C1-Ee) to ~ 11.95 Ma (ACM/C4-Cp), where only *Micromeryx* morphotype 2 is recorded. Overall, *Micromeryx* maintained sharp apices, high-relief cusp and low average MS of 0.1, and tooth enamel δ^13^C values of − 12.0 ± 0.3‰ (VPDB) (Table 1) that point to the consumption of plant resources from relatively dense wooded areas (see Additional file 1: Supplementary information, Note 4 for explanation of the calculated δ^13^C cut-off values among different habitats). Estimated MAP ranges from 928 to 1190 mm/year (depending on whether a correction for altitude and latitude is applied or not) (Table 1). In phase I, *Micromeryx* tooth enamel δ^18^O_CO3_ and δ^18^O_PO4_ values are the highest among the three environmental phases, with calculated MAT values reaching 21.8 °C (Table 1). According to Whittaker’s biome classification [50], estimated MAP and MAT for phase I would correspond to those of a tropical seasonal forest/savanna (Additional file 5: Figure S4). This agrees with the soft-leafy browsing diet inferred from mesowear and indicates a humid climate with rainfall seasonality (although not marked) and the development of long-standing forests with bushy and woody vegetation [23, 31, 51]. This type of environment at the beginning of the ACM series, characterised by humid and warm forests with a dense upper canopy, is somewhat more seasonal than previous inferences for ACM as a whole [52] and would be suitable for the multiple large-bodied hominoids—*A. brevirostris*, *D. fontani* and *P. catalaunicus*—recorded during phase I. The abundance of trees may have allowed hominoids to eat a diverse array of vegetation, ranging from leaves and soft fruits (*Anoiapithecus* and *Dryopithecus*) to harder and brittle fruits (*Pierolapithecus*) [11, 41]. This also fits with the postcranial morphology of *Pierolapithecus*, which indicates an orthograde bodyplan with adaptations for arboreal vertical climbing [10, 21, 34, 53]. Only a single *Micromeryx* specimen from ACM/C2-A3 (IPS29396) displays more rounded cusps and slightly higher δ^13^C and δ^18^O values. This specimen might reflect the exploitation of more abrasive elements probably located along less humid—but still forested—patches in specific localities. It is noteworthy that only one pliopithecoid is recorded in this first phase (Fig. 3 (a)) (Pliopithecoidea indet. from ACM/C3-B2 at 12.06 Ma). This immediately precedes a first short pulse of decreased humidity as documented by ACM/C2-A3, which might explain the lack of great ape record between their first appearance in the sequence at 12.4–12.3 and their more abundant record at 12.0–11.9 [5]. This shifting climatic pattern at 11.98 Ma toward less humid conditions might have also influenced (preferred) food availability (as seen in *Micromeryx*) and impelled hominoids to exploit alternative sources, especially as fallback foods [41], not consumed before.

#### Phase II

ACM localities experienced a different environmental phase from ~ 11.90 to 11.79 Ma. There was a rapid increase in *Micromeryx* phenotypic diversity and population abundance after ACM/C3-Ak (11.88 Ma), with the first co-occurrence of all morphotypes (at least three) being recorded at ACM/C4-C1 (Fig. 3 (b–d)). Compared to phase I, from 11.88 Ma onwards the less sharp and more rounded cusp shapes, higher average MS of 0.16 of *Micromeryx* and the increase in the mean δ^13^C (− 11.4 ± 1.2‰, VPDB) (Table 1, Fig. 3 (b, c)) are consistent with less humid and more open areas during this part of the ACM sequence. The broader range of δ^13^C observed in phase II (Fig. 3 (c)) is congruent with a phase of increased habitat heterogeneity. Estimated MAP ranges from 765 to 992 mm/year, whereas estimated MAT is 20.3 °C (Table 1). The biomes of phase II would be in the domains of tropical seasonal forest/savanna and subtropical desert [50] (Additional file 5: Figure S4). In the light of the fauna recorded at ACM [15], we consider the latter inference as unrealistic and most likely attributable to a preservational bias toward drier ecosystems [54]. Alternatively, higher CO_2_ levels during the Miocene might produce a similar bias in biome reconstructions, given their documented relationship not only with higher temperatures but also enhanced water-use efficiency and leaf-level productivity [55]. This “forest fertilization effect”, resulting from higher CO_2_ levels in the Miocene, might have resulted in more forested environments than indicated by estimated MAP and TAP based on current standards. Discerning whether such potential biases apply uniformly to the whole ACM sequence would require a taxonomically broader isotopic sampling in selected ACM localities—as averaging values from multiple taxa from the same locality would arguably provide more robust MAP estimates [54]. Nevertheless, we consider that the palaeoenviromental changes recorded by *Micromeryx* isotopic values through time are at least valid in relative terms, even if their exact interpretation in terms of extant biomes should be subject to further scrutiny. The development of mosaic environments (with the earlier forested habitats containing for the first time partial clearing as new open patches) in ACM might have allowed the local evolution of new *Micromeryx* morphotypes (i.e. species) adapted to more open landscapes and with different dietary preferences (e.g. more abrasive forbs, shrubs and other ligneous vegetation rich in phytoliths, and even some grass). Such an interpretation is reinforced by the record at ACM/C3-Ak of the bovid *Tethytragus*, a common faunal element in the more open and arid palaeoenvironments from inner Iberia that is otherwise not documented from the Vallès-Penedès Basin [56]. Our results support greater habitat heterogeneity, rather than a complete change in the palaeoenvironment compared to the previous phase. On the one hand, *Micromeryx* morphotype 2 (with affinity for humid conditions) persists in phase II with little variation in MS and δ^13^C—with higher values at the end of phase II (at ACM/C4-C1, 11.81 Ma) likely indicating that more abrasive foods and/or some grit loaded foliage was eaten at this time. On the other hand, the new morphotypes 1 and 3 appear for the first time with higher mean MS and δ^13^C values (Fig. 3 (b, c)).

These changes toward habitat (canopy) fragmentation, leading to a mosaic of forest patches interrupted by more open woodlands and maybe even shrublands, would have represented a challenge for the frugivorous and presumably arboreal great apes from ACM—especially in dietary terms (given the impossibility of maintaining a year-round supply of ripe fruits), and perhaps also from a locomotor viewpoint (at least for the highly arboreal *Pierolapithecus*, given the need to travel across the canopy to exploit such resources). Admittedly, arboreal adaptations are only unambiguously recorded for the orthograde *Pierolapithecus* [10, 21, 34, 53], while there are no postcranials for *Anoiapithecus*, and *Dryopithecus* appears to have been more pronograde [57]. Nevertheless, there are no indications that *Dryopithecus* was particularly adapted to terrestrial locomotion [57], whereas in contrast *Pliopithecus* has been inferred as a generalised semiarboreal quadruped [58, 59], which would have allowed the latter genus to better exploit not only the shrub level but also to walk terrestrially among different arboreal feeding sources. Furthermore, the smaller body size of pliopithecoids would have likely allowed them to exploit smaller patches of fruiting trees than in the case of great apes. Overall, the inferred palaeoenvironmental changes might explain why great apes became even rarer faunal elements and were eventually replaced by pliopithecoids during this phase. Our results therefore differ from those of previous analyses based on hypsodonty [4], according to which pliopithecoids generally inhabited more humid environments than hominoids. This discrepancy is largely attributable to the different methods used. The work by Sukselainen et al. [4] was devised to detect general patterns over broader geographical (all Eurasia) and temporal (most of the Miocene) scales than our study, which is designed to test such patterns in a small region with a shorter time span (ca. 1 Myr) but with considerably higher temporal resolution. The environmental constraints affecting the occurrence of the different primate taxa may vary between distant geographic regions and at different time intervals. For example, the findings by Sukselainen et al. [4] are certainly influenced by the inclusion of several hominoid taxa (such as *Kenyapithecus*, *Ankarapithecus* and *Ouranopithecus*) that surely lived in more arid habitats than pliopithecoids [3], which may result in the overestimation of some subtle (but real) differences between hominoids and pliopithecoids. Accordingly, our conclusions cannot be generalised to Miocene European primates as a whole. To test this possibility, similar analyses focused on other pliopithecoid and hominoid species from elsewhere would be required.

#### Phase III

Our results provide compelling evidence for a distinct phase toward the top of the ACM sequence (11.70–11.60 Ma), roughly coinciding with the middle to late Miocene boundary. During such a short interval, dryopithecines reappear at 11.65 Ma after a gap of > 200 kyr in which only *Pliopithecus* was recorded, and overlap with the small-bodied stem hominoid *Pliobates* (not previously recorded), even coexisting in a particular locality (ACM/C5-D1; 11.63 Ma [5]). *Micromeryx* morphotypes yield MS higher on average than in phase II (average MS of 0.32), less sharp and more rounded cusps, and—for the first time in the sequence—low occlusal relief (Table 1, Fig. 3 (b)). *Micromeryx* tooth enamel mean δ^13^C (− 11.1 ± 1.2‰, VPDB) (Fig. 3 (c)) is also higher. This evidence lends support to increased opening of the canopy, with trees growing in more open conditions or sparser formations. MS and δ^13^C ranges are the widest (up to twice that recorded at any other previous phase) of the sequence, which is consistent with greater habitat heterogeneity than in phase II. Phase III witnesses the lowest δ^18^O_CO3_ and δ^18^O_PO4_ values (Table 1, Fig. 3 (d)). Calculated MAP reaches the lowest value of the three phases ranging from 608 to 901 mm/year, and estimated MAT is also the lowest inferred with 17.1 °C—which means a drop of 3 °C compared to phase II (Table 1, Fig. 3 (c, d)). All this evidence suggests a woodland/shrubland biome [50] (Additional file 5: Figure S4). Nonetheless, the record of the aquaphilous tragulid *Dorcatherium naui* [60], flying squirrels [61] and arboreal dormice at ACM/C5-D1 indicate that permanent water masses (palustrine areas or streams) and forests were also present in this phase. Accordingly, our results are consistent with more open, mosaic environments in phase III, although they may have been interspersed with restricted gallery forests, which would have provided a denser tree cover and preserved some subtropical plant elements—contrasting with the predominant vegetation of the nearby areas. Such kind of habitats would have probably been suitable for *Pliobates* (whose small body size would have enabled the exploitation of smaller areas of fruiting trees), which displays a mosaic of primitive and derived postcranial features consistent with cautious and eclectic arboreal climbing, and even some degree of suspensory behaviours [11]—as opposed to the more generalised semiterrestrial quadrupedalism of *Pliopithecus* mentioned above. The decrease in estimated MAP and MAT values throughout the three-phased sequence proposed in this study may be tracking the progressive drop in temperatures that took place throughout the middle and late Miocene and that was responsible for biome change in the Iberian Peninsula, from subtropical to drier and more open conditions [48, 49].

### The rare co-occurrence of pliopithecoids and hominoids

Combining tooth wear patterns and stable isotope data for the moschid *Micromeryx* allows us to reconstruct for the first time the local palaeoenvironmental changes that took place during the ca. 1 Myr time span recorded by the ACM series and provide insight on the factors underpinning the rare co-occurrence of hominoids and pliopithecoids. Our results conclusively show that ACM habitats significantly changed with the passing of time and that the recorded turnovers in the primate faunal assemblage are correlated with three main successive environmental phases. Humid conditions are only predominantly present during phase I (at the lowermost portion of the sequence, 12.38–11.95 Ma), where most large-bodied and presumably arboreal hominoids are recorded; from then onwards, humid ecosystems are only intermittently present in the area and only the smaller-bodied and presumably semiterrestrial pliopithecoids are recorded during phase II. Hominoid-bearing localities thus emerge as different from those recording pliopithecoids, with the former corresponding to quite homogeneous habitats with high humidity and continuous canopies (i.e. densely wooded environments). In contrast, pliopithecoid-bearing localities correspond to less humid and more patchy habitats with a reduced canopy cover. Dryopithecines are recorded again, together with the small-bodied arboreal hominoid *Pliobates*, during the third phase (corresponding to the uppermost portion of the sequence; 11.70–11.60 Ma), characterised by more open conditions and increased environmental heterogeneity. This might be explained by the existence of narrow forest settings (with more humid conditions than its surroundings), to which hominoids would have probably been restricted to a large extent.

## Conclusions

Our results indicate that the chronostratigraphic distribution of pliopithecoids and hominoids, as previously suspected [1, 4, 5], is not merely attributable to sampling biases, as the various primate taxa are consistently present in different types of environments. The purported preference of pliopithecoids for more humid environments as compared to large-bodied hominoids, previously inferred based on hypsodonty data [4], is not supported in the case of ACM. More generally, our results clearly show that hominoid and pliopithecoids exploited different environments, and support that they displayed differences in behavioural ecology, largely “tracking” the most suitable habitats for each group—which would explain why they only co-occurred at a few localities throughout the European Miocene. Our results further support the view that ACM hominoids, like other Miocene apes as a whole, inhabited more seasonal and arid environments than extant apes [3]. Indeed, isotopic data for the Siwaliks (Pakistan) based on some particular herbivorous mammals (not including *Micromeryx*) [62, 63] and rodents show higher d^13^C values than those of the Vallès-Penedès [64], which evidences that *Sivapithecus*, which is present at some of those sites, might have inhabited somewhat drier environments. However, given the potential biases of the record, additional mesowear and isotopic analyses comprising a wider array of taxa would be required to provide more refined palaeoenvironmental reconstructions for specific ACM localities.

Furthermore, the results of our study illustrate that continuous and densely sampled fossiliferous sequences, such as that from ACM, are essential for deciphering the complex interplay between biotic and abiotic factors that shape the chronostratigraphic and geographic distribution patterns of past taxa. Even if local, such sequences enable removing potential biases caused by differences in geographical situation as well as local physiographic and climatic factors among localities in continental-wide studies. In other words, densely sampled sequences permit detailed focus on the temporal component of palaeoenvironmental change and, hence, arguably have a greater potential to test hypotheses on the correlation between faunal, climate and habitat change at a much finer scale than studies at a more global scale. Extending such local comparisons to other densely sampled sequences covering similar time spans elsewhere in Eurasia would be the logical next step to further investigate the general validity of the conclusions drawn in this paper for the rare co-occurrence of hominoids and pliopithecoids as a whole.

## Methods

### Hypothesis-testing framework

We proceed with the null hypothesis that the chronostratigraphic distribution of pliopithecoids and hominoids through ACM is random relative to the palaeoenvironmental conditions inferred from the associated fauna. If the null hypothesis cannot be rejected, it would follow that hominoids and pliopithecoids might have exploited the same (even if varied) habitats and, hence, the possibility that their rare co-occurrence is merely a statistical artefact due to insufficient sampling could not be discounted. If the null hypothesis is rejected, because one group is consistently recorded in different types of environments, then it would follow that their rare co-occurrence is likely attributable to different ecological preferences.

### Data set and ACM stratigraphic series

*Micromeryx* (Additional file 1: Supplementary information, Note 1 and Additional file 2: Figure S1) specimens analysed in this paper are housed in the Institut Català de Paleontologia Miquel Crusafont (ICP) in Sabadell (Spain). They come from 11 fossil localities in the 250-m-thick ACM composite stratigraphic sequence [5, 15], in els Hostalets de Pierola (Vallès-Penedès Basin, Catalonia, Spain; Fig. 1a–c). These localities are restricted areas corresponding to a single layer (or stratigraphic horizon), usually between 0.2 and 1.0 m in thickness, from which fossil remains were collected (see Alba et al. [5] for further details). The sequence includes more than 200 micro- and/or macromammal localities and spans more than 1 Myr (12.6 to 11.4 Ma; late Aragonian, middle to late Miocene, MN7+8) [5]. The genus *Micromeryx*, widely dispersed across Europe and ubiquitous [18], is common throughout the whole ACM sequence, thus allowing for a stratigraphically continuous sampling. The oldest occurrence of *Micromeryx* sp. in the sequence corresponds to the locality ACM/C1-Ee, with an estimated age of 12.38 Ma, whereas the youngest occurrence corresponds to ACM/C5-D1 (11.63 Ma) (Fig. 1c). The remaining investigated ACM localities cluster in an interval spanning from 11.68 to 12.23 Ma (Fig. 1c). We analyse patterns of tooth wear and enamel stable isotope composition—including carbon (δ^13^C) and oxygen (δ^18^O_CO3_ and δ^18^O_PO4_)—of three different *Micromeryx* morphotypes (termed morphotypes 1, 2 and 3). They represent three distinct species, although we refrain from formally naming them (see more details in Additional file 1: Supplementary information, Note 1, Additional file 3: Figure S2 and Additional file 4: Figure S3).

The use of a single moschid genus in palaeoecological analysis constitutes a novelty of this study, as this group has not previously been explored from the standpoint of mesowear and stable isotopes in tandem, due to the small size of its teeth and the consequent difficulty in gathering the data. Combining both approaches allows us to make robust inferences not only about the feeding ecology of *Micromeryx*, but also in estimating climatic and environmental parameters through the ACM sequence. Mesowear and stable isotopes represent different time spans of the animal’s life and operate over different temporal scales—with the former recording an average of the diet during the last years before death [25, 65] and the latter corresponding to the time period of tooth mineralisation, which usually records earlier years of the animal’s ontogeny [66]. When inferring palaeoenvironmental change though time (in relation to temporal changes in the composition of the primate assemblage), focusing the study on *Micromeryx* minimises the biases that would be introduced by analysing a wider array of taxa, which are much less ubiquitously and continuously recorded across the ACM sequence. Indeed, adding more taxa could contribute to refining palaeoenvironmental reconstructions for particular sites and stratigraphic bins, but it would also introduce biases caused by the sparser record of less abundant taxa along the ACM sequence.

### Long-term patterns of tooth mesowear

Mesowear is a method for determining diet (browse vs. graze) and abrasiveness of foods and exogenous items based on gross dental wear [26]. It provides an immediate long-term dietary signal compared to the individual’s lifespan and is a very powerful tool for documenting changes in fossil habitats and climate [27, 51]. Mesowear for 45 teeth, each one belonging to a different individual, was recorded. The young and old adult individuals were identified by dental wear and discarded from analyses [67]. Preference was given to upper cheek teeth (usually second molars) but lower teeth were also evaluated when necessary following Fraser et al. [68]. Individual cusp shape and occlusal relief were scored on the buccal cups. Cusp shape was categorised as either sharp = pS, rounded = pR, or blunt = pB, according to the degree of facet development. Occlusal relief was categorised as high = pH or low= pL, depending on the relative difference in height between the tip of the cusp and the intercusp valley. A mesowear score (MS) for each ACM locality was obtained in order to trace temporal evolution following a five-point scoring system [69]: 0 = high relief and sharp cusps; 1 = high relief and rounded cusps; 2 = low relief and rounded cusps; 2.5 = low relief and sharp cusps; and 3 = low relief and blunt cusps. We do not use the more recent scoring system by Mihlbachler et al. [70] because it was specifically devised for the analysis of equid mesowear. Although the method could potentially work with other taxa, we prefer to rely on the scoring system [69] customarily used to score ruminant mesowear. See Additional file 1: Supplementary information, Note 2 for further details on this method.

### Stable isotope analysis

Stable carbon and oxygen isotope compositions (δ^13^C and δ^18^O) of mammalian tooth enamel reflect diet, primary production and animal and landscape water use, which permit to reconstruct palaeoecological preferences and, in turn, past environmental and climatic conditions [24, 66, 71, 72]. For herbivorous terrestrial mammals, tooth enamel δ^13^C values are largely controlled by the photosynthetic pathway followed by the ingested plants, as well as by plant water economy, and inform about the vegetation cover present in an area [73]. The δ^18^O values of mammalian bioapatite—both the carbonate fraction (δ^18^O_CO3_) and the phosphate fraction (δ^18^O_PO4_)—record the δ^18^O value of body water, which in turn reflects the fluxes and δ^18^O value of oxygen entering and exiting the body. Within an obligate-drinking taxon, variations in body water δ^18^O values can be interpreted as chiefly reflecting changes in the isotopic composition of ingested water, which varies with mean annual temperature and aridity [24, 66, 71, 72].

A total of 37 samples were analysed for δ^13^C and δ^18^O_CO3_, whereas 30 tooth enamel samples were analysed for δ^18^O_PO4_. Tooth enamel was sampled using a rotary drill with a diamond-tipped dental burr under a microscope to recover enamel from an area of the tooth as large as possible to avoid seasonal bias in the time of mineralisation. Sampling protocol and chemical treatment follows Domingo et al. [24] for carbonate and phosphate in bioapatite.

When performing isotopic analyses on fossil material, diagenetic effects must be addressed before attempting to extract palaeoecological, palaeoenvironmental and/or palaeoclimatic information. When both δ^18^O_CO3_ and δ^18^O_PO4_ values have been determined, post mortem alteration can be checked by calculating the difference between δ^18^O_CO3_ and δ^18^O_PO4_ (∆^18^O_CO3_-_PO4_ = δ^18^O_CO3_ − δ^18^O_PO4_). This follows the premise that bioapatite in modern mammals have a restricted difference of ~ 8.6–9.1‰ [22, 23]. ∆^18^O_CO3_-_PO4_ mean value for the whole dataset of *Micromeryx* samples is 8.6 ± 0.8‰, and therefore, it is within the expected range for unaltered bioapatite.

Mean annual precipitation (MAP) from *Micromeryx* tooth enamel δ^13^C was estimated following Kohn [54], both with and without altitude and latitude correction. Estimated mean annual temperature (MAT) was also calculated following a two-stepped method and using published equations [74, 75]. See Additional file 1: Supplementary information, Notes 3 and 4, for a full description of sample processing and chemical treatment, and estimated MAP and MAT calculations.

### Statistical analyses

Chi-square (*χ*^2^) and Fisher’s exact tests with level of significance *p* < 0.05 were performed based on individual mesowear score for testing for significant differences in mesowear between the three *Micromeryx* morphotypes. Shapiro-Wilk tests and Q-Q plots revealed normal distributions for isotopic data. Parametric analysis of variance (ANOVA) and Turkey’s post hoc pairwise comparisons were performed to test for significant mean differences at *p* < 0.05 between all possible pairs using a Studentised range distribution. Locally weighted polynomial lines were fitted to the isotopic data. Calculations for isotopic data further include a 95% confidence region. Descriptive and inferential statistics were conducted using R software version 3.5.1.

## Supplementary Information

**Additional file 1.** Additional information on the moschid *Micromeryx*, tooth-wear patterns and stable isotope analysis.

**Additional file 2: Figure S1.** *Moschus* and *Micromeryx*. A *Moschus moschiferus* adult male. Image by Vladimir Prikhod’ko, reproduced with permission of the author. B Skull of *M. moschiferus* showing the enlarged upper canines of males. C MPZ 2006/413, skull of a juvenile male *Micromeryx azanzae* from Toril-3 (middle Miocene, MN7+8, Zaragoza, Spain). D Life reconstruction of a *Micromeryx* male. Art by Mauricio Antón, reproduced with permission of the author.

**Additional file 3: Figure S2.** ACM *Micromeryx*, examples of the lower dentition of the morphotypes with remarks on their most conspicuous features. A Morphotype 1, specimens IPS90767 and IPS43689. B Morphotype 2, specimen IPS44457. C Morphotype 3, specimens IPS43907 and IPS57264.

**Additional file 4: Figure S3.** ACM *Micromeryx*, examples of the upper dentition of the morphotypes with remarks on their most conspicuous features. A Morphotype 1, specimens IPS44358 and IPS45524. B Morphotype 2, specimens IPS45552, IPS48103 and IPS29766. C Morphotype 3, specimen IPS45382. Anatomical definitions: protocone-T, T-shaped fold of enamel formed at the terminal end of the post-protocrista; metaconule-T, T-shaped fold of enamel formed at the terminal end of the pre-metaconulecrista.

**Additional file 5: Figure S4.** Whittaker’s biome classification plot showing terrestrial biome-types aas function of mean annual precipitation (MAP; in cm) and temperature (MAT; in °C). ACM localities from the three distinct environmental phases are plotted according to calculated values of estimated MAP and MAT using average stable carbon and oxygen isotope values of *Micromeryx* enamel apatite. For each locality we show estimated MAP before (circles) and after (squares) correcting for palaeolatitude and palaeoaltitude. Note that these corrections result in lower estimates. Diagram modified from Whittaker [42].

**Additional file 6: Table S1.** Individual values of mesowear and stable isotopes of *Micromeryx* specimens from the Abocador de Can Mata stratigraphic sequence (Vallès-Penedès Basin).

**Additional file 7: Table S2.** ANOVA and Tukey post hoc tests for δ^13^C (‰ VPDB), δ^18^O_CO3_ (‰ VSMOW), and δ^18^O_PO4_ (‰ VSMOW) values of *Micromeryx* from Abocador de Can Mata according to morphotypes and environmental phases. Values in bold are statistically significant (*p* < 0.05).

## Data Availability

All data generated or analysed during this study are included in this published article and its additional information files.
