# Publisher Correction to: Palaeoecological differences underlie rare co-occurrence of Miocene European primates

**DOI:** 10.1186/s12915-021-01089-y

**Published:** 2021-07-30

**Authors:** Daniel DeMiguel, Laura Domingo, Israel M. Sánchez, Isaac Casanovas-Vilar, Josep M. Robles, David M. Alba

**Affiliations:** 1 ARAID foundation / Universidad de Zaragoza, Departamento de Ciencias de la Tierra, and Instituto Universitario de Investigación en Ciencias Ambientales de Aragón (IUCA), Pedro Cerbuna 12, 50009 Zaragoza, Spain; 2 Institut Català de Paleontologia Miquel Crusafont, Universitat Autònoma de Barcelona, Edifici ICTA-ICP, C/ Columnes s/n, Campus de la UAB, 08193 Cerdanyola del Vallès, Barcelona, Spain; 3 Departamento de Geodinámica, Estratigrafía y Paleontología Facultad de Ciencias Geológicas, Universidad Complutense de Madrid, José Antonio Novais 12, 28040 Madrid, Spain; 4 Earth and Planetary Sciences Department, University of California Santa Cruz, 1156 Hight Street, Santa Cruz, CA 95064 USA

**Publisher Correction to:** ***BMC Biology*** **19, 6 (2021).**

10.1186/s12915-020-00939-5

Following publication of the original article [1], the authors noted that an incorrect version of Additional file 1 was published. The corrected version of Additional file 1 is attached to this Publisher Correction and the Additional file 1 has been updated in the original article.

The publisher apologises to the authors and readers for the inconvenience caused by the error.

## Supplementary Information

**Additional file 1:** Additional information on the moschid Micromeryx, tooth-wear patterns and stable isotope analysis.
