# Supplementary material for: Palaeoecological differences underlie rare co-occurrence of Miocene European primates
Source: BMC Biol. 2021 Jan 19;19:6. doi: 10.1186/s12915-020-00939-5 (PMC7814646; doi:10.1186/s12915-020-00939-5)
Supplement: Supplementary file 1 — Additional file 1. Additional information on the moschid Micromeryx, tooth-wear patterns and stable isotope analysis. [file 12915_2020_939_MOESM1_ESM.pdf]

Supplementary Information for

Palaeoecological differences underlie rare co-occurrence of Miocene European primates

DeMiguel et al.

Table of Contents

- **Supplementary Note 1**: The moschid *Micromeryx* and its taxonomic assignment at ACM
- **Supplementary Note 2**: Tooth-wear patterns
- **Supplementary Note 3**: Stable isotope analysis
- **Supplementary Note 4**: Mean Annual Precipitation (MAP) and Mean Annual Temperature (MAT) calculation

- **References**

Supplementary Note 1: The moschid *Micromeryx* and its taxonomic assignment at ACM

Moschids (musk-deer) are hornless bovoid pecorans [1,2] only represented today by a single genus, *Moschus* (Fig. S1A, B), with seven described species that inhabit the mountainous areas of Asia, ranging from Vietnam to Pakistan[3]. Small-sized, strange and secretive, moschids once comprised one of the most successful Miocene ruminant clades. They are first recorded from MN4 deposits (ca. 17 Ma) [4] and during the next 5 million years constituted a common component of the Eurasian continental mammalian faunas, with a palaeobiogeographical distribution that stretched from East Asia to the Iberian Peninsula [1,4,5-11]. They became extinct in Europe ca. 8 Ma, being subsequently restricted to Asia. In Iberia they inhabited almost every kind of continental biome, from more or less open savannas to (sub)tropical forests [5,8].

Among Miocene moschids, *Micromeryx* (Fig. S1C, D) is the most diverse and the one with a more marked disparity both in morphology and size [6,7,10,11]. *Micromeryx* has been recovered as the sister group to extant *Moschus* (Sánchez et al., 2010, 2015). In Iberia, it was abundant and diverse, with two endemic species (*M. azanzae* and *M. soriae*) besides the nominotypical *M. flourensianus* [5-7] and additional species yet to be described (I.M.S. unpublished data; see also below).

In the Abocador de Can Mata (ACM) sequence, *Micromeryx* is by far the most ubiquitous and best-represented large mammal taxon. I.M.S. and D.DM. identified six types of dentition (three lower + three upper) that correspond to three dental morphotypes (Figs. S2 and S3) and, accordingly, to three species that we leave unassigned to genus as *Micromeryx* sp., until future systematic analyses allow us to refine their taxonomic attribution. The three identified morphotypes do not seem to correspond to either subspecies or variants of a single polymorphic species of *Micromeryx*, but to three distinct species. Morphotype 2 represents a derived *Micromeryx* species of *Micromeryx soriae* or *Micromeryx mirus* type, with corresponding sets of derived dental morphology in both upper and lower series. Morphotype 3 represents a more plesiomorphic *Micromeryx* species, with broad cusps/cuspids, narrow inner valleys and several other corresponding features. Finally, morphotype 1 represents an ‘intermediate’ species,

similar in some aspects to *M. azanzae* (Fig. S1C). The three identified morphotypes are characterised by the following features:

Morphotype 1

Lower dentition. p4 with long posterolabial cristid; mesial cingulid much less developed than in morphotype 3; reduced metastylid; and lack of *Palaeomeryx*-fold. Like morphotype 3, this morphotype is less derived than 2 and larger than morphotype 3. (Fig. S2A).

Upper dentition. Square upper molars with a large distal lobe, including the M3 (which lacks the reduction of the distal lobe typical of this tooth position and displays instead a well-developed distal lobe and the same square occlusal contour as the preceding molars); more or less developed metaconule-T; developed entostyle (more related to the metaconule than to the protocone), parastyle and mesostyle; developed metastyle in the M3. Morphotype 1 can also have a very long metaconule-fold that almost forms a fossete. Also, the P4 shows a buccal-fold in the distolingual crista that can be so developed that it forms a small fossa (Fig. S3A).

Morphotype 2

Lower dentition. Relatively high crowns, similar to *M. soriae* or *M. mirus*, with buccolingually compressed cuspids; lingual elements relatively less marked than in morphotypes 1 and 3; variable presence of *Palaeomeryx*-fold branching from the protoconid; long distolabial cristid in the p3; lingually interupted cristid obliqua that almost reaches the posterolingual conid, and long posterolabial cristid in the p4; similarly sized premetacristid and preprotocristid, producing a mesial V-shape morphology; large ectostylid and marked metastylid; m3 with mesially located and compressed lingual cuspid of the third lobe; mesial cingulid with well-marked lingual and buccal parts, but less developed central part (Fig. S2B).

Upper dentition. Protocone-T that is often well developed; more or less flat cusps; presence of well-developed metaconule-fold; more or less parallel and narrow styles and buccal structures, resembling the morphology of derived *Micromeryx* with higher molar crowns (e.g.,

M. mirus and *M. soriae*); and well-developed entostyles that are located in a middle position between the protocone and the metaconule (Fig. S3B).

Morphotype 3.

Lower dentition. Cuspids with broader bases, producing very narrow inner valleys; short distal stylids in the p3-p4; short and well-marked metastylid reduced only to the final tip, button-type; strong *Paleomeryx*-fold branching off from the protoconid; m3 with distally closed third lobe, located in a central position, and with a large buccal cuspid, almost as large as the second lobe; poorly developed cristids; robust lingual elements; well-developed and rounded ectostylid; and small mesial cingulid (Fig. S2C). This is the most plesiomorphic morphotype.

Upper dentition. Protocone-T that is usually small, although the presence of this structure is variable; molars with broad main cusps with inner-facing orientation that result in narrow inner valleys; styles and buccal structures that are broad and more similar to those of morphotype 1; small ectostyle always linked to the base of the protocone; metaconule-fold that can extend to almost form a pit; and accessory small pits formed by enamel folds that can be present at the centre of the molars (Fig. S3C).

Supplementary Note 2: Tooth-wear patterns

The use of mesowear and stable isotope analyses on fossils are methods of palaeoecological analyses that allow to infer the specific diet of individuals (independently from adaptation) as well as to determine climatic and environmental conditions. They are the most valuable tools allowing comparison of biomes and ecological changes through time in response to shifts in local environments [12-18]. Here we rely on these techniques in tandem with the high-resolution stratigraphic sequence of Abocador de Can Mata.

Dental mesowear is considered a good dietary proxy in herbivorous large-mammals, as it reflects the cumulative wear imposed on teeth during a relatively long period of feeding activity as a consequence of both the abrasive elements intrinsic to plants themselves and exogenous grit encroaching on food items [19]. Mesowear relied originally only on second upper molars (M2),

but was later extended to all upper positions [20]. To avoid any bias due to postmortem damage, teeth were screened under a stereomicroscope to identify and discard those specimens with poor preservation of occlusal enamel surfaces and taphonomical alterations [21].

Dental cusp shape (sharp, rounded or blunt) and occlusal relief (high or low) were examined for 45 specimens with the naked eye and using a low-magnification (10×) stereomicroscope Olympus SZ11 and qualitatively scored by D.DM. Mesowear score (MS) for each fossil locality was obtained according to Rivals et al. [22]. Data of mesowear for modern taxa allow to calculate a range of MS and transition among dietary preferences (D.DM. unpublished data): values from 0 to 0.2 usually correspond to soft, C_3-dominated diets; values from 0.6 to 1.1 to intermediate C_3-C_4 diets; and values higher than 1.4 to abrasive, C_4-dominated diets. Note that are transitional values (0.2 to 0.6 and 1.1 to 1.4) in where taxa with different diets overlap.

Supplementary Note 3: Stable isotope analysis

Carbon ($\delta^{13}C$) and oxygen ($\delta^{18}O_{CO3}$ and $\delta^{18}O_{PO4}$) stable isotope composition of *Micromeryx* sp. in tooth enamel was analysed by L.D. Tooth enamel was sampled using a rotary drill with a diamond-tipped dental burr under a microscope to recover enamel from an area of the tooth as large as possible to avoid seasonal bias in the time of mineralization. Carbon and oxygen isotope results are reported in δ-notation $\delta^{H}X_{sample} = [(R_{sample}–R_{standard})/R_{standard}] \times 1000$, where X is the element, H is the mass of the rare, heavy isotope, and R=$^{13}C/^{12}C$ or $^{18}O/^{16}O$. Vienna Pee Dee Belemnite (VPDB) is the standard for $\delta^{13}C$ values, and $\delta^{18}O$ values are reported relative to Vienna Standard Mean Ocean Water (VSMOW). Samples (N = 37) were analysed for the carbon and oxygen isotope composition of carbonate in bioapatite ($\delta^{13}C$ and $\delta^{18}O_{CO3}$, respectively). Carbonate analyses were conducted at the stable isotope laboratory of the University of California Santa Cruz using a ThermoScientific MAT253 dual inlet isotope ratio mass spectrometer coupled to a ThermoScientific Kiel IV carbonate device. Approximately 2–3 mg of tooth enamel were sampled and treated with 30% H_2O_2 for 24 h. Samples were rinsed 5 times in deionised (DI) water and soaked for 24 h in 1 M acetic acid buffered to ~ pH 5 using

Ca acetate solution. After 5 rinses with DI water, the resulting solid was freeze-dried at -40 °C and at a pressure of 25×10^{-3} Mbar for 24 h. In order to remove any trace of water within the treated bioapatite powder samples that may affect $\delta^{18}O$ values, they were roasted at 65 °C under vacuum for one hour and a half before IRMS analysis. The standards used were Carrara Marble (CM, $\delta^{13}C$ = 2.05‰ and $\delta^{18}O$ = -1.91‰), NBS-18 ($\delta^{13}C$ = -5.03‰ and $\delta^{18}O$ = -23.01‰) and NBS-19 ($\delta^{13}C$ = 1.95‰ and $\delta^{18}O$ = -2.20‰). The standard deviations for repeated measurements of CM (N = 12), NBS-18 (N = 8) and NBS-19 (N = 4) were 0.02‰, 0.02‰ and 0.03‰ for $\delta^{13}C$, respectively, and 0.10‰, 0.16‰ and 0.11‰ for $\delta^{18}O$, respectively. Duplicate analyses were carried out for ~20% of the samples (N = 7). The average absolute differences for $\delta^{13}C$ and $\delta^{18}O_{CO3}$ values were 0.02‰ and 0.13‰, respectively, and the standard deviations of these average differences were 0.01‰ and 0.09‰ for $\delta^{13}C$ and $\delta^{18}O_{CO3}$ values, respectively. Mass loss after pretreatment of samples for the analyses of the carbonate fraction of tooth enamel was imperceptible and post-treatment yield was nearly 100% in all cases. This is due to the practical inexistence of organic matter (removed through the use of H_2O_2) and diagenetic, secondary or external carbonate (removed through the use of acetic acid buffered with Ca acetate) present in the selected *Micromeryx* tooth enamel.

The oxygen isotope composition of phosphate in bioapatite ($\delta^{18}O_{PO4}$) was measured on 30 enamel samples. Analyses were performed at the stable isotope laboratory of the University of California Santa Cruz using a ThermoScientific Delta plus XP continuous flow isotope ratio mass spectrometer coupled to a ThermoFinnigan High Temperature Conversion Elemental Analyser (TCEA). The chemical treatment is described in Bassett et al. [23]. Between 1.5 and 2 mg of tooth enamel were recovered and dissolved in 100 µl of 0.5 M HNO_3. 75 µl of 0.5 M KOH and 200 µl of 0.36 M KF were added to neutralise the solution and to precipitate CaF_2 and other fluorides, respectively. Samples were then centrifuged and after removing the resulting solid, 250 µl of silver amine solution (0.2 M $AgNO_3$, 0.35 M NH_4NO_3, 0.74 M NH_4OH) was added and the samples were maintained at 50 °C overnight to precipitate Ag_3PO_4. The resulting Ag_3PO_4 crystals were recovered by centrifugation and rinsing with DI water (5 times), after

which vials were placed in an oven overnight at 50 °C. The standards used were Fisher standard ($\delta^{18}O$ = 8.4‰), UCSC High standard ($\delta^{18}O$ = 19.0‰), UCSC Low standard ($\delta^{18}O$ = 11.3‰) and NIST 120c ($\delta^{18}O$ = 21.8‰). The standard deviations for repeated measurements of Fisher Standard (n = 14), UCSC High standard (N = 4), UCSC Low standard (N = 3) and NIST 120c (N = 4) were 0.31‰, 0.14‰, 0.59‰ and 0.34‰, respectively. Duplicate $\delta^{18}O_{PO4}$ analyses were carried out on ~ 60% of the samples. The average absolute difference for $\delta^{18}O_{PO4}$ was 0.08‰ and the standard deviation of this average difference was 0.21‰. Mass loss after pretreatment of samples for the analyses of the phosphate fraction of tooth enamel was of ~60%. This is within the expected range as the phosphate protocol does not just entail removal of organic matter and external carbonates (as is the case of the carbonate protocol). The chemical treatment of the bioapatite phosphate fraction implies an actual conversion to a different chemical compound (from hydroxyapatite to silver phosphate), in a way that the TCEA can break the chemical bonds to exclusively analyse the oxygen within the PO_4^{-3} ion.

The working temperature of the chemical pretreatment for both, the carbonate and phosphate fractions of bioapatite was room temperature with the exception of those steps that required the use of an oven.

Supplementary Note 4: Mean Annual Precipitation (MAP) and Mean Annual Temperature (MAT) calculation

Carbon isotope ($\delta^{13}C$) composition analysed on mammalian bioapatite constitutes an excellent tool to characterize ancient dietary ecology [24]. For herbivorous mammals, it is largely controlled in terrestrial settings by the photosynthetic pathway followed by the consumed plants [25-29]. C_3 plants (trees, shrubs, forbs and cool-season grasses) strongly discriminate against ^{13}C during CO_2 fixation. $\delta^{13}C$ values for these plants range from -36‰ to -22‰, with an average value of -27‰. C_4 plants (grasses and sedges from areas with a warm growing season and some arid-adapted dicots) discriminate less against ^{13}C, yielding $\delta^{13}C$ values that vary between -17‰ to -9‰, with a mean value of -13‰. A third photosynthetic

pathway, the Crassulacean Acid Metabolism (CAM) occurs mainly in succulent plants and displays intermediate isotopic values between C_3 and C_4 plants. Nevertheless, CAM plants were not presumably abundant during the middle Miocene at the Vallès-Penedès Basin and, moreover, moschids are unlikely to have exploited this resource, had it been present. Therefore, we will not consider CAM plants in our study. Studies carried out on modern ruminants have shown that $\delta^{13}C$ values of mammalian tooth enamel ($\delta^{13}C_{enamel}$) track the $\delta^{13}C$ values of the plants they consume ($\delta^{13}C_{diet}$), with an offset of ca. +14.1±0.5‰ due to fractionation linked to carbonate equilibria and metabolic processes [30,31]. If we assume that this fractionation value obtained for modern ruminants can be applied to extinct taxa, we can estimate cut-off $\delta^{13}C$ values to distinguish among different habitats. However, when focusing on the fossil record, it is necessary to consider to what extent the atmospheric $\delta^{13}C$ values ($\delta^{13}C_{atm}$) have varied with respect to present values. Anthropogenic modification due to fossil fuel burning have given rise to a decrease in $\delta^{13}C_{atm}$ values from -6.5‰ to -8‰ since the onset of the Industrial Revolution [32,33]. $\delta^{13}C_{atm}$ has also witnessed natural oscillations throughout Earth history. Following $\delta^{13}C_{atm}$ values estimated for the Cenozoic based on benthic foraminiferal records [34], for the time interval considered in this study (~12.4–11.6 Ma) we take a $\delta^{13}C_{atm}$ value of -6‰. Accounting for a 2‰ offset with respect to modern $\delta^{13}C_{atm}$ baseline, the $\delta^{13}C$ threshold between C_3-dominated diet and intermediate C_3-C_4 diet is set between ~ -9‰ and -8‰, whereas the $\delta^{13}C$ threshold between intermediate C_3-C_4 diet and C_4-dominated diet is set between ~ -2‰ and -1‰.

Mean Annual Precipitation (MAP) was calculated following Kohn [35]. First a modern equivalent of diet composition ($\delta^{13}C_{diet,\ meq}$) is calculated following the equation:

$$\delta^{13}C_{diet,\ meq} = \delta^{13}C_{leaf} + (\delta^{13}C_{modern\ atm\ CO2} - \delta^{13}C_{ancient\ atm\ CO2}) \quad (1)$$

where $\delta^{13}C_{leaf}$ is $\delta^{13}C_{tooth}$ – 14.1‰ (Cerling and Harris, 1999), $\delta^{13}C_{modern\ atm\ CO2}$ is -8‰, and $\delta^{13}C_{ancient\ atm\ CO2}$ is the mean $\delta^{13}C_{atmCO2}$ value from Tipple et al. [34].

Then MAP is calculated by resolving the following equation by Kohn [35]:

$$\delta^{13}C_{diet,\ meq} = -10.29 + 1.90 \times 10^{-4}\ \text{Altitude (m)} - 5.61\log_{10}(\text{MAP} + 300,\ \text{mm/yr}) - 0.0124\text{Abs (latitude, °)} \quad (2)$$

MAP was calculated 1) without correcting for altitude and latitude, and 2) with altitude = 200 m and latitude = 41 °N, which are conservative estimated values for the ACM area in the middle to late Miocene.

Oxygen isotope ($\delta^{18}O$) composition of both carbonate ($\delta^{18}O_{CO3}$) and phosphate ($\delta^{18}O_{PO4}$) fractions of mammalian tooth enamel reflect $\delta^{18}O$ of body water ($\delta^{18}O_{bw}$), which in turn records oxygen uptake (inspired O_2 and water vapor, drinking water, dietary water, oxygen in food dry matter) and loss (excretion, expired CO_2 and water vapor) during tooth development [35,36]. Mammals can be classified, depending on their water requirements, in obligate drinkers (those that obtain most of their water from drinking) and non-obligate drinkers (those that obtain water mainly from plant water and metabolic water). In a similar way, different African mammals were classified, based on their isotopic sensitivity to aridity, as evaporation insensitive (EI) and evaporation sensitive (ES) [37]. EI mammals are those that drink water on a daily basis or consume non-leafy parts of plants (containing water not subject to evaporation) and record local $\delta^{18}O$ meteoric values. Therefore, changes associated with $\delta^{18}O_{bw}$ values of EI mammals mirror variations in the isotopic composition of ingested (meteoric) water, which is in turn positively correlated with Mean Annual Temperature (MAT) and aridity. ES mammals are those that survive with little or no drinking water, obtaining most of their water from leafy parts of plants, which is more prone to strong evaporative ^{18}O-enrichment. In these animals, oxygen isotope composition of bioapatite will be partly determined by carbohydrates, lipids and protein from their diet, which will produce metabolic water [35]. When considering extinct mammals such as *Micromeryx*, it is difficult to assess the type of water economy they may have had. Nowadays, the family Moschidae is represented by a single genus, *Moschus*, which occupies forested and mountainous areas of Asia [3,38]. Although extant musk deer rely on a browsing diet during the summer and lichen-based diet during the winter, it has been observed that they drink water on a daily basis (0.5-0.8 L of daily water intake in the summer at 20 °C; Prikhod'ko, pers. comm.),

such that, presumably, their tooth enamel $\delta^{18}O$ signal will be largely dependent on ingested water $\delta^{18}O$ values. Following this premise and assuming a similar water dependence for its sister taxon *Micromeryx*, we have calculated MAT from *Micromeryx* tooth enamel $\delta^{18}O_{PO4}$ values to obtain information about the thermal variability throughout the temporal ACM sequence. MAT was calculated following a two-stepped method. First, the oxygen isotope composition of the body water ($\delta^{18}O_{bw}$)—which represents the oxygen isotope composition of meteoric water ($\delta^{18}O_{mw}$) in obligate drinkers or EI mammals—was worked out from *Micromeryx* tooth enamel $\delta^{18}O_{PO4}$ values using the following equation for modern ruminants [39]:

$$\delta^{18}O_{mw(VSMOW)} = (\delta^{18}O_{PO4(VSMOW)} - 25.53) / 1.13 \quad (3)$$

$\delta^{18}O_{mw}$ was corrected for the change in ocean oxygen isotope composition between today and the middle to late Miocene by subtracting 0.3 ‰ from the calculated $\delta^{18}O_{mw}$ value [40]. Then, we used a regression equation between MAT and $\delta^{18}O_{mw}$ calculated from published meteorological data [41]:

$$MAT\ (°C\ ) = \delta^{18}O_{mw(VSMOW)} + 12.68/ 0.36 \quad (4)$$

This equation is preferred against those calculated for modern Iberian areas close to the ACM sequence because during the middle to late Miocene, climatic regimes were very different from extant ones. As the equation above was built upon a global meteorological dataset [41], including different climatic regimes, it produces a more averaged MAT value with less biases due to the excessive weight of a single climatic zone. Once that MAP and MAT values were calculated for ACM localities, possible palaeobiomes were inferred following Whittaker´s biome classification [42] (Fig. S4).

References

1. Sánchez, IM, Domingo, MS, Morales, J. The genus *Hispanomeryx* (Mammalia, Ruminantia, Moschidae) and its bearing on musk-deer phylogeny and systematics. Palaeontology. 2010;53:1023–1047.
2. Sánchez, IM, Cantalapiedra, JL, Ríos, M, Quiralte, V, Morales, J. Systematics and evolution of the Miocene three-horned palaeomerycid ruminants (Mammalia, Cetartiodactyla). PLoS ONE. 2015;10:e0143034.
3. Groves, C.P. in *Handbook of the Mammals of the World*. *Volume 2: Hoofed Mammals* 2011 (Lynx Editions).
4. Radovic, P, Alaburic, S. in *Life on the Shore: Geological and Paleontological Research the Neogene of Sibnica and Vicinity (Lebac Basin, Central Serbia), Part 1* 2016;141-148 (Special Issue of the Natural History Museum, Belgrade, Serbia).
5. Sánchez, IM, Morales, J. Distribución biocronológica de los Moschidae (Mammalia; Ruminantia) en España. Estud Geol. 2006;62:533–546.
6. Sánchez, IM, Morales, J. *Micromeryx azanzae* sp. nov. (Ruminantia: Moschidae) from the middle-upper Miocene of Spain, and the first description of the cranium of *Micromeryx*. J Vertebr Paleontol. 2008;28:873–885.
7. Sánchez, IM, Domingo, MS, Morales, J. New data on the Moschidae (Mammalia, Ruminantia) from the upper Miocene of Spain. J Vertebr Paleontol. 2009;29:567–575.
8. Sánchez, IM, DeMiguel, D, Almécija, S, Moyà-Solà, S, Morales, J, Alba, DM. New *Hispanomeryx* (Mammalia, Ruminantia, Moschidae) from Spain and a reassessment of the systematics and paleobiology of the genus *Hispanomeryx* Morales, Moyà-Solà and Soria, 1981. J Vertebr Paleontol. 2019;39:e1602536.
9. Wang, S, Shi, Q, Hui, Z, Li, Y, Zhang, J, Peng, T. Diversity of the Moschidae (Ruminantia, Artiodactyla, Mammalia) in the middle Miocene of China. Paleontol Res. 2015;19:143–155.
10. Aiglstorfer, M, Mayda, S, Heizmann, E. First record of late middle Miocene Moschidae from Turkey: *Micromeryx* and *Hispanomeryx* from Catakbağyaka (Muğla, SW Turkey). C R Palevol. 2018;17:178–188.

11. Aiglstorfer, M, Costeur, L, Mennecart, B, Heizmann, EPJ. *Micromeryx*? *eiselei*–a new moschid species from Steinheim am Albuch, Germany, and the first comprehensive description of moschid cranial material from the Miocene of Central Europe. PLoS ONE. 2017;12:e0185679.
12. Domingo, L, Koch, PL, Grimes, ST, Morales, J, López-Martínez, N. Isotopic paleoecology of mammals and the Middle Miocene Cooling event in the Madrid Basin (Spain). Palaeogeogr Palaeoclimatol Palaeoecol. 2012;339–341:98–113.
13. Domingo, L, Koch, PL, Hernández Fernández, M, Fox, D, Domingo, MS, Alberdi, MT. Late Neogene and Early Quaternary paleoenvironmental and paleoclimatic conditions in Southwestern Europe: isotopic analyses on ungulate taxa. PLoS ONE. 2013;8:e63739.
14. Domingo, L, Domingo, MS, Koch, PL, Morales, J, Alberdi, MT. Carnivoran resource and habitat use in the context of a Late Miocene faunal turnover episode. Palaeontology. 2017;60:461–483.
15. DeMiguel, D, Azanza, B, Morales J. Trophic flexibility within the oldest Cervidae lineage to persist through the Miocene Climatic Optimum. Palaeogeogr Palaeoclimatol Palaeoecol. 2010;289:81–92.
16. DeMiguel, D, Azanza, B, Morales J. Paleoenvironments and paleoclimate of the Middle Miocene of central Spain: A reconstruction from dental wear of ruminants. Palaeogeogr Palaeoclimatol Palaeoecol. 2011;302:452–463.
17. DeMiguel, D, Rook, L. Understanding climate's influence on the extinction of *Oreopithecus* (late Miocene, Tusco-Sardinian paleobioprovince, Italy). J Hum Evol. 2018;116:14–26.
18. DeMiguel, D, Azanza, B, Morales J. Regional impacts of global climate change: A local humid phase in central Iberia in a late Miocene drying world. Palaeontology. 2019;62:77–92.
19. Fortelius, M, Solounias, N. Functional characterization of ungulate molars using the abrasion-attrition wear gradient: a new method for reconstructing paleodiets. Am Mus Novit. 2000;3301:1–35.
20. Kaiser, TM, Solounias, N. Extending the tooth mesowear method to extinct and extant equids. Geodiversitas. 2003;25:321–345.

21. DeMiguel, D. (2016). Disentangling adaptive evolutionary radiations and the role of diet in promoting diversification on islands. Sci. Rep. *6*, 29803.
22. Rivals, F, Schulz, E, Kaiser, TM. Late and middle Pleistocene ungulates dietary diversity in Western Europe indicate variations of Neanderthal paleoenvironments through time and space. Quat Sci Rev. 2009;28:3388–3400.
23. Bassett, D, MacLeod, KG, Miller, JF, Ethington, RL. Oxygen isotopic composition of biogenic phosphate and the temperature of Early Ordovician seawater. Palaios. 2007;22:98–103.
24. Koch, PL. in *Stable Isotopes in Ecology and Environmental Science* 2007;99–154 (Blackwell Publishing, Boston).
25. Bender, MM. Variations in the $^{13}C/^{12}C$ ratios of plants in relation to the pathway of photosynthetic carbon dioxide fixation. Phytochemistry. 1971;10:1239–1245.
26. O'Leary, MH. Carbon isotopes in photosynthesis. BioScience. 1988;38:328–336.
27. Farquhar, GD., Ehleringer, JR., Hubick, KT. Carbon isotopic discrimination and photosynthesis. Annu. Rev. Plant Physiol. Plant Mol Biol. 1989;40:503–537.
28. Ehleringer, JR., Monson, RK. Evolutionary and ecological aspects of photosynthetic pathway variation. Ann Rev Ecol Evol System. 1993;24:411–439.
29. Hayes, JM. Fractionation of carbon and hydrogen isotopes in biosynthetic processes. Rev Mineral Geochem. 2001;43:225–277.
30. Cerling, TE, Harris, JM. Carbon isotope fractionation between diet and bioapatite in ungulate mammals and implications for ecological and paleoecological studies. Oecologia, 1999;120:347–363.
31. Passey, BH. et al. Carbon isotope fractionation between diet, breath CO_2, and bioapatite in different mammals. J Archaeol Sci. 2005;32:1459–1470.
32. Friedli, H, Lotscher, H, Oeschger, H, Siegenthaler, U, Stauver, B. Ice core record of the $^{13}C/^{12}C$ ratio of atmospheric CO_2 in the past two centuries. Nature. 1986;324:237–238.
33. Marino, BD, McElroy, MB. Isotopic composition of atmospheric CO_2 inferred from carbon in C_4 plant cellulose. Nature. 1991;349:127–131.

34. Tipple, BJ, Meyers, SR, Pagani, M. Carbon isotope ratio of cenozoic CO_2: a comparative evaluation of available geochemical proxies. Paleoceanography. 2010;25:PA3202.
35. Kohn, MJ. Predicting animal $\delta^{18}O$: accounting for diet and physiological adaptation. Geochim Cosmochim Acta. 1996;60:4811–4829.
36. Bryant, JD, Froelich, PN. A model of oxygen isotope fractionation in body water of large mammals. Geochim Cosmochim Acta. 1995;59:4523–4537.
37. Levin, NE, Cerling, TE, Passey, BH, Harris, JM, Ehleringer, JR. A stable isotope aridity index for terrestrial environments. Proc Natl Acad Sci USA. 2006;103:11201–11205.
38. Prikhod'ko, VI. *Musk Deer. Origins, Taxomomy, Ecology, Behaviour and Communication* 2003 (GEOS, Moscow).
39. D'Angela, D, Longinelli, A. Oxygen isotopes in living mammal's bone phosphate: Further results. Chem Geol. 1990;86:75–82.
40. Lear, CH, Elderfield, H, Wilson, PA. Cenozoic deep-sea temperatures and global ice volumes from Mg/Ca in benthic foraminiferal calcite. Science. 2000;287: 269–272.
41. Rozanski, K, Araguás-Araguás, L, Gonfiantini, R. in *Climate change in continental isotopic records* 1993;1–36 (American Geophysical Union).
42. Whittaker, RH. Classification of natural communities. Bot Rev. 1962;28:1–239.
43. Kohn, MJ. Carbon isotope compositions of terrestrial C_3 plants as indicators of (paleo)ecology and (paleo)climate. Proc Natl Acad Sci USA. 2010;107:19691–19695.
