# Supplementary material for: Palaeoecological differences underlie rare co-occurrence of Miocene European primates
Source: BMC Biol. 2021 Jan 19;19:6. doi: 10.1186/s12915-020-00939-5 (PMC7814646; doi:10.1186/s12915-020-00939-5)
Supplement: Supplementary file 2 — Additional file 2: Figure S1. Moschus and Micromeryx. A Moschus moschiferus adult male. Image by Vladimir Prikhod’ko, reproduced with permission of the author. B Skull of M. moschiferus showing the enlarged upper canines of males. C MPZ 2006/413, skull of a juvenile male Micromeryx azanzae from Toril-3 (middle Miocene, MN7+8, Zaragoza, Spain). D Life reconstruction of a Micromeryx male. Art by Mauricio Antón, reproduced with permission of the author. [file 12915_2020_939_MOESM2_ESM.pdf]

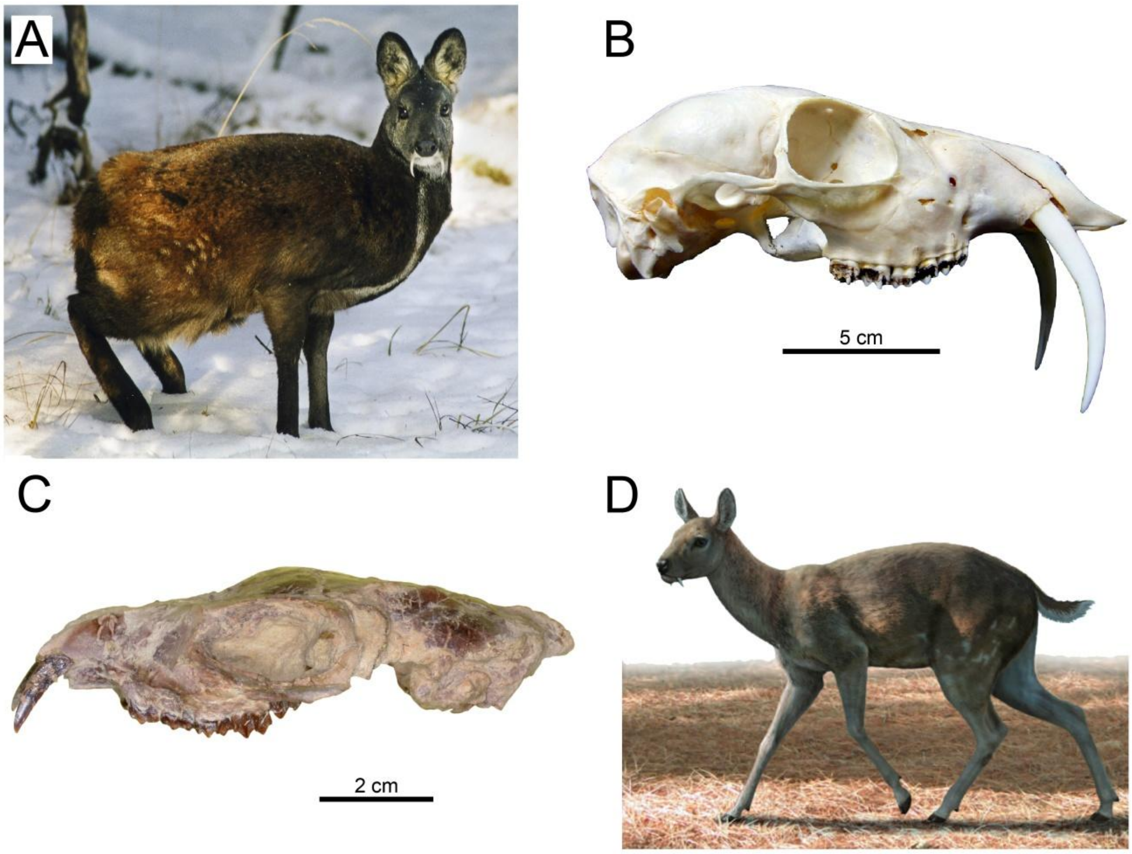

Figure S1. ***Moschus* and *Micromeryx***. **A** *Moschus moschiferus* adult male. Image by Vladimir Prikhod'ko, reproduced with permission of the author. **B** Skull of *M. moschiferus* showing the enlarged upper canines of males. **C** MPZ 2006/413, skull of a juvenile male *Micromeryx azanzae* from Toril-3 (middle Miocene, MN7+8, Zaragoza, Spain). **D** Life reconstruction of a *Micromeryx* male. Art by Mauricio Antón, reproduced with permission of the author.
