# Supplementary material for: Palaeoecological differences underlie rare co-occurrence of Miocene European primates
Source: BMC Biol. 2021 Jan 19;19:6. doi: 10.1186/s12915-020-00939-5 (PMC7814646; doi:10.1186/s12915-020-00939-5)
Supplement: Supplementary file 3 — Additional file 3: Figure S2. ACM Micromeryx, examples of the lower dentition of the morphotypes with remarks on their most conspicuous features. A Morphotype 1, specimens IPS90767 and IPS43689. B Morphotype 2, specimen IPS44457. C Morphotype 3, specimens IPS43907 and IPS57264. [file 12915_2020_939_MOESM3_ESM.pdf]

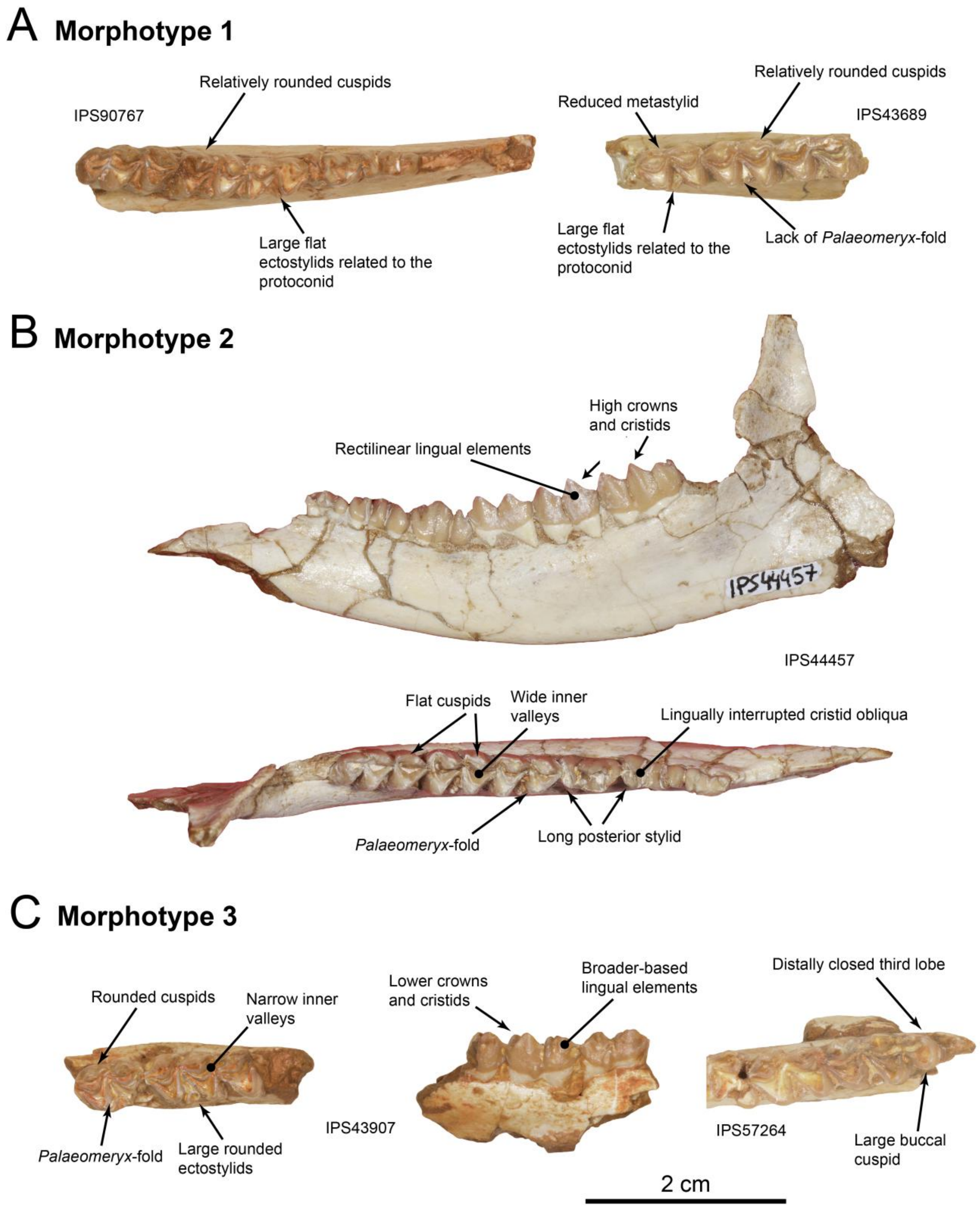

Figure S2. ACM *Micromeryx*, examples of the lower dentition of the morphotypes with remarks on their most conspicuous features. **A** Morphotype 1, specimens IPS90767 and IPS43689. **B** Morphotype 2, specimen IPS44457. **C** Morphotype 3, specimens IPS43907 and IPS57264.
