# Supplementary material for: Palaeoecological differences underlie rare co-occurrence of Miocene European primates
Source: BMC Biol. 2021 Jan 19;19:6. doi: 10.1186/s12915-020-00939-5 (PMC7814646; doi:10.1186/s12915-020-00939-5)
Supplement: Supplementary file 4 — Additional file 4: Figure S3. ACM Micromeryx, examples of the upper dentition of the morphotypes with remarks on their most conspicuous features. A Morphotype 1, specimens IPS44358 and IPS45524. B Morphotype 2, specimens IPS45552, IPS48103 and IPS29766. C Morphotype 3, specimen IPS45382. Anatomical definitions: protocone-T, T-shaped fold of enamel formed at the terminal end of the post-protocrista; metaconule-T, T-shaped fold of enamel formed at the terminal end of the pre-metaconulecrista. [file 12915_2020_939_MOESM4_ESM.pdf]

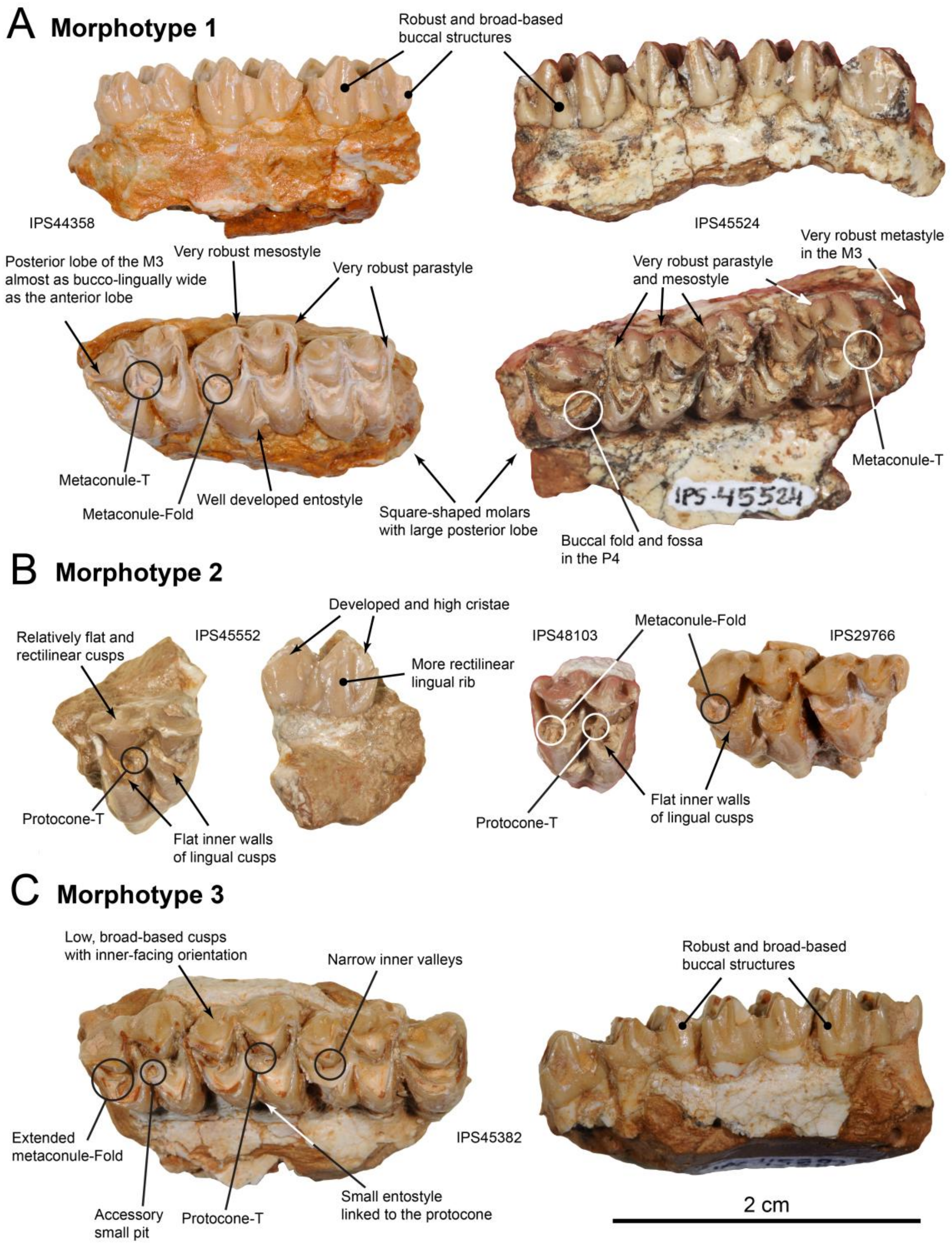

Figure S3. ACM *Micromeryx*, examples of the upper dentition of the morphotypes with remarks on their most conspicuous features. **A** Morphotype 1, specimens IPS44358 and IPS45524. **B** Morphotype 2, specimens IPS45552, IPS48103 and IPS29766. **C** Morphotype 3, specimen IPS45382. Anatomical definitions: protocone-T, T-shaped fold of enamel formed at the terminal end of the post-protocrista; metaconule-T, T-shaped fold of enamel formed at the terminal end of the pre-metaconulecrista.
