# Supplementary material for: Palaeoecological differences underlie rare co-occurrence of Miocene European primates
Source: BMC Biol. 2021 Jan 19;19:6. doi: 10.1186/s12915-020-00939-5 (PMC7814646; doi:10.1186/s12915-020-00939-5)
Supplement: Supplementary file 5 — Additional file 5: Figure S4. Whittaker’s biome classification plot showing terrestrial biome-types aas function of mean annual precipitation (MAP; in cm) and temperature (MAT; in °C). ACM localities from the three distinct environmental phases are plotted according to calculated values of estimated MAP and MAT using average stable carbon and oxygen isotope values of Micromeryx enamel apatite. For each locality we show estimated MAP before (circles) and after (squares) correcting for palaeolatitude and palaeoaltitude. Note that these corrections result in lower estimates. Diagram modified from Whittaker [42]. [file 12915_2020_939_MOESM5_ESM.pdf]

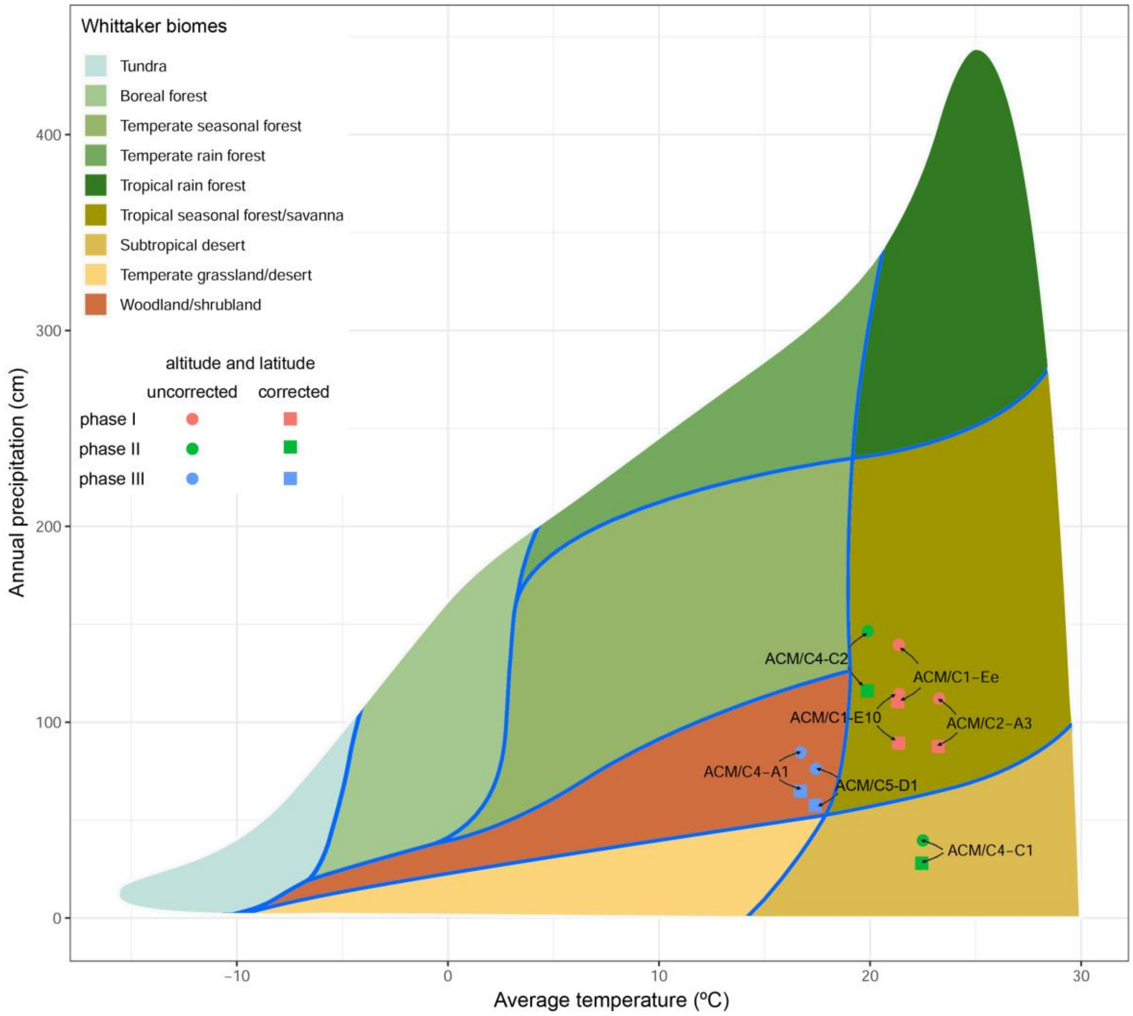

Figure S4. Whittaker's biome classification plot showing terrestrial biome-types aas function of mean annual precipitation (MAP; in cm) and temperature (MAT; in °C). ACM localities from the three distinct environmental phases are plotted according to calculated values of estimated MAP and MAT using average stable carbon and oxygen isotope values of *Micromeryx* enamel apatite. For each locality we show estimated MAP before (circles) and after (squares) correcting for palaeolatitude and palaeoaltitude. Note that these corrections result in lower estimates. Diagram modified from Whittaker (1962).
