# Supplementary material for: Palaeoecological differences underlie rare co-occurrence of Miocene European primates
Source: BMC Biol. 2021 Jan 19;19:6. doi: 10.1186/s12915-020-00939-5 (PMC7814646; doi:10.1186/s12915-020-00939-5)
Supplement: Supplementary file 6 — Additional file 6: Table S1. Individual values of mesowear and stable isotopes of Micromeryx specimens from the Abocador de Can Mata stratigraphic sequence (Vallès-Penedès Basin). [file 12915_2020_939_MOESM6_ESM.pdf]

Table S1. Individual values of mesowear and stable isotopes of *Micromeryx* specimens from the Abocador de Can Mata stratigraphic sequence (Vallès-Penedès Basin).

Locality	Age (Ma)	Label	Species	Morphotype	OR	OR	CS	CS	$\delta^{13}C_{CO3}$ (‰ VPDB)	$\delta^{18}O_{CO3}$ (‰ VPDB)	$\delta^{18}O_{CO3}$ (‰ VSMOW)	$\delta^{18}O_{PO4}$ (‰ VSMOW)	$\Delta\ \delta^{18}O_{CO3}$-$\delta^{18}O_{PO4}$	$\delta^{13}C_{diet}$ (‰ VPDB)	$\delta^{13}C_{diet.\ mequ}$ (‰ VPDB)	MAP^a	MAP^b	MAT
ACM/C5-D1	11.63	IPS45349	*Micromeryx*	2	—	h	—	r	-10.2	-5.8	25.0	16.7	8.3	-24.3	-26.3	414,8	289.3	12,6
ACM/C5-D1	11.63	IPS45511	*Micromeryx*	3	h	h	—	s	-12.2	-3.6	27.2	18.4	8.8	-26.3	-28.3	1309	1026	16.8
ACM/C5-D1	11.63	IPS43934	*Micromeryx*	3	h	h	s	s	-10.6	-3.6	27.2	17.7	9.4	-24.7	-26.6	520.9	376,8	15,2
ACM/C5-D1	11.63	IPS48106	*Micromeryx*	1	h	h	s	r	—	—	—	—	—	—	—	—	—	—
ACM/C5-D1	11.63	IPS48111	*Micromeryx*	1	h	h	s	r	-11.0	-3.0	27.9	19.5	8.3	-25.1	-27.1	676	505	19.5
ACM/C5-D1	11.63	IPS44829	*Micromeryx*	3	h	h	s	s	-10.4	-4.5	26.3	17.3	8.9	-24.5	-26.4	448.9	317	14.2
ACM/C5-D1	11.63	IPS48122	*Micromeryx*	2	—	h	—	r	-11.1	-3.4	27.4	18.5	8.9	-25.2	-27.2	718.9	540	17
ACM/C5-D1	11.63	IPS48127	*Micromeryx*	1	—	—	—	—	-10.3	-1.4	29.4	21.5	8.0	-24.4	-26.4	446.2	315	24.3
ACM/C5-D1	11.63	IPS48132	*Micromeryx*	1	h	h	s	—	—	—	—	—	—	—	—	—	—	—
ACM/C5-D1	11.63	IPS45355	*Micromeryx*	2	h	h	s	s	-10.4	-3.6	27.2	18.5	8.7	-24.5	-26.5	469.6	334	17
ACM/C5-D1	11.63	IPS45748	*Micromeryx*	2	h	h	—	—	-12.6	-1.4	29.4	20.5	9.0	-26.7	-28.6	1548	1223	21.8
ACM/C5-D1	11.63	IPS48105	*Micromeryx*	3	h	h	s	r	—	—	—	—	—	—	—	—	—	—
ACM/C5-D1	11.63	IPS48108	*Micromeryx*	3	h	h	s	s	—	—	—	—	—	—	—	—	—	—
ACM/C5-D1	11.63	IPS44386	*Micromeryx*	3	l	—	r	—	—	—	—	—	—	—	—	—	—	—
ACM/C5-D1	11.63	IPS45727/IPS48108	*Micromeryx*	2	—	h	—	s	-11.8	-3.2	27.6	18.0	9.6	-25.9	-27.9	1066	826	15.9

ACM/C4-A1	11.70	IPS29542	*Micromeryx*	2	—	—	—	—	-11.2	-1.8	29.1	20.7	8.3	-25.3	-27.3	780.0	591	22.5
ACM/C4-A1	11.70	IPS46539	*Micromeryx*	2	—	—	—	—	-12.7	-4.8	26.0	17.7	8.3	-26.8	-28.7	1629	1290	15.1
ACM/C4-A1	11.70	IPS46708	*Micromeryx*	3	—	h	s	—	—	—	—	—	—	—	—	—	—	—
ACM/C4-A1	11.70	IPS47726	*Micromeryx*	1	—	—	—	—	-11.5	-3.4	27.4	18.7	8.7	-25.6	-27.6	899.2	689	17.5
ACM/C4-A1	11.70	IPS29631	*Micromeryx*	1	h	h	r	s	-9.6	-2.8	28.0	19.7	8.3	-23.7	-25.7	258.1	160	19.9
ACM/C4-A1	11.70	IPS45841/IPS45844	*Micromeryx*	1	h	h	s	s	-11.2	-3.9	26.9	17.7	9.2	-25.3	-27.3	779	590	15.1
ACM/C4-A1	11.70	IPS29634	*Micromeryx*	2	—	h	—	s	-10.7	-5.4	25.3	16.1	9.2	-24.8	-26.8	563.4	412	11.3
ACM/C4-A1	11.70	IPS71616	*Micromeryx*	2	—	h	—	r	—	—	—	—	—	—	—	—	—	—
ACM/C4-A1	11.70	IPS46709	*Micromeryx*	2	—	h	—	s	-10.9	-4.4	26.4	15.4	11.0	-25.0	-27.0	637.9	473	9.4
ACM/C4-A1	11.70	IPS46718	*Micromeryx*	2	h	h	r	s	—	—	—	—	—	—	—	—	—	—
ACM/C4-A1	11.70	IPS45842/IPS47616	*Micromeryx*	3	h	h	—	—	-10.5	-4.2	26.6	20.1	6.4	-24.6	-26.6	508.4	366	21.1
ACM/C4-A1	11.70	IPS47628	*Micromeryx*	3	h	h	s	s	-12.6	-3.9	26.9	19.1	7.8	-26.7	-28.6	1553	1227	18.5
ACM/C5-C2	11.79	IPS43689	*Micromeryx*	1	h	h	s	r	-10.1	-4.3	26.5	—	—	-24.2	-26.1	366	249	—
ACM/C4-C1	11.81	IPS29743	*Micromeryx*	2	—	h	—	s	-10.8	-4.0	26.7	—	—	-24.9	-26.9	613.1	453	—
ACM/C4-C1	11.81	IPS47300	*Micromeryx*	2	h	h	—	r	—	—	—	—	—	—	—	—	—	—
ACM/C4-C1	11.81	IPS47303	*Micromeryx*	3	h	h	s	s	-9.5	-2.8	28.0	—	—	-23.6	-25.5	217.5	127	—
ACM/C4-C1	11.81	IPS32980	*Micromeryx*	1	h	h	s	—	-10.3	-2.3	28.6	20.7	7.8	-24.4	-26.4	446.2	315	22.5
ACM/C4-C1	11.81	IPS47302	*Micromeryx*	1	h	h	—	s	-9.8	-2.4	28.4	—	—	-23.9	-25.9	308.3	201	—
ACM/C5-C3	11.86	IPS57291	*Micromeryx*	3	h	h	—	—	—	—	—	—	—	—	—	—	—	—

ACM/C3-Ak	11.88	IPS63414	*Micromeryx*	2	h	h	s	—	—	—	—	—	—	—	—	—	—	—
ACM/C3-Ak	11.88	IPS46109	*Micromeryx*	2	h	h	s	s	—	—	—	—	—	—	—	—	—	—
ACM/C3-Ak	11.88	IPS57264	*Micromeryx*	unknown	h	h	r	—	-11.0	-1.6	29.2	—	—	-25.1	-27.1	683.4	511	—
ACM/C4-C2	11.90	IPS43699	*Micromeryx*	2	—	—	—	—	-12.9	-3.8	26.9	—	—	-27.0	-28.9	1799	1430	—
ACM/C4-C2	11.90	IPS43590	*Micromeryx*	2	—	—	—	—	-12.8	-2.8	28.0	20.2	7.8	-26.9	-28.8	1733	1376	21.1
ACM/C4-C2	11.90	IPS49661	*Micromeryx*	2	h	h	—	s	-12.2	-0.9	29.9	21.9	8.0	-26.3	-28.2	1268	993	25.4
ACM/C4-C2	11.90	IPS44427	*Micromeryx*	2	h	—	s	—	-13.1	-4.1	26.7	17.6	9.1	-27.2	-29.2	2010	1605	14.8
ACM/C4-C2	11.90	IPS42233	*Micromeryx*	2	h	h	s	s	-11.5	-2.1	28.7	20.7	8.0	-25.6	-27.5	874	668	22.5
ACM/C4-C2	11.90	IPS42367	*Micromeryx*	2	—	h	—	s	-12.5	-4.3	26.4	17.9	8.5	-26.6	-28.5	1498	1183	15.6
ACM/C4-C2	11.90	IPS42185	*Micromeryx*	unknown	—	h	—	s	-11.8	-4.3	26.5	—	—	-25.9	-27.9	1073	832	—
ACM/C4-Cp	11.95	IPS63421	*Micromeryx*	2	h	h	s	s	—	—	—	—	—	—	—	—	—	—
ACM/C2-A3	11.98	IPS29396	*Micromeryx*	2	h	h	s	r	-11.9	-1.3	29.6	21.0	8.6	-26.0	-28.0	1121	871	23.3
ACM/C2-A3	11.98	IPS29407	*Micromeryx*	2	h	h	—	s	—	—	—	—	—	—	—	—	—	—
ACM/C1-E10	12.28	IPS30004	*Micromeryx*	2	h	h	s	s	-12.1	-2.4	28.5	19.6	8.9	-26.2	-28.2	1257	984	19.7
ACM/C1-E10	12.28	IPS30000	*Micromeryx*	2	h	h	—	s	-11.7	-1.0	29.8	21.6	8.3	-25.8	-27.7	985	759	24.6
ACM/C1-E10	12.28	IPS33105	*Micromeryx*	2	h	h	s	s	-12.0	-3.1	27.8	19.7	8.1	-26.1	-28.1	1192	930	19.9
ACM/C1-Ee	12.38	IPS29405	*Micromeryx*	2	h	h	s	—	-12.3	-2.0	28.8	20.3	8.6	-26.4	-28.4	1395	1097	21.4

Individual occlusal relief (OR) scored as high (h) and low (l); cusp shape (CS) scored as sharp (s), and rounded (r); $\delta^{13}C$ (‰ VPDB); $\delta^{18}O_{CO3}$ (‰VPDB); $\delta^{18}O_{CO3}$ (‰ VSMOW); $\delta^{18}O_{PO4}$ (‰ VSMOW); $\Delta\delta^{18}O_{CO3}$-$\delta^{18}O_{PO4}$; $\delta^{13}C_{diet}$ (‰ VPDB); $\delta^{13}C_{diet, mequ}$ (‰ VPDB); inferred mean MAP (mm/yr) (from Kohn 2010) without (MAP[a]) and with (MAP[b]) altitude and latitude correction; and inferred mean MAT (°C).
