# Supplementary material for: Palaeoecological differences underlie rare co-occurrence of Miocene European primates
Source: BMC Biol. 2021 Jan 19;19:6. doi: 10.1186/s12915-020-00939-5 (PMC7814646; doi:10.1186/s12915-020-00939-5)
Supplement: Supplementary file 7 — Additional file 7: Table S2. ANOVA and Tukey post hoc tests for δ13C (‰ VPDB), δ18OCO3 (‰ VSMOW), and δ18OPO4 (‰ VSMOW) values of Micromeryx from Abocador de Can Mata according to morphotypes and environmental phases. Values in bold are statistically significant (p < 0.05). [file 12915_2020_939_MOESM7_ESM.pdf]

Table S2. ANOVA and Tukey post hoc tests for δ^{13}C (‰ VPDB), $\delta^{18}O_{CO3}$ (‰ VSMOW), and $\delta^{18}O_{PO4}$ (‰ VSMOW) values of *Micromeryx* from Abocador de Can Mata according to morphotypes and environmental phases. Values in bold are statistically significant ($p < 0.05$).

Morphotype			Environmental phases		
**δ^{13}C values** (F = 6.949, df =2, **p = 0.003**)	Morphotype 2	Morphotype 3	δ^{13}C values (F = 1.639, df =2, p = 0.209)	Phase II	Phase III
Morphotype 1	**0.004**	0.609	Phase I	0.463	0.187
Morphotype 2		0.117	Phase II		0.733
$\delta^{18}O_{CO3}$ values (F = 0.845, df =2, p = 0.439)	Morphotype 2	Morphotype 3	**$\delta^{18}O_{CO3}$ values** (F = 4.175, df =2, **p = 0.024**)	Phase II	Phase III
Morphotype 1	0.927	0.438	Phase I	0.158	**0.019**
Morphotype 2		0.511	Phase II		0.443
$\delta^{18}O_{PO4}$ values (F = 0.562, df =2, p = 0.577)	Morphotype 2	Morphotype 3	**$\delta^{18}O_{PO4}$ values** (F = 3.977, df =2, **p = 0.031**)	Phase II	Phase III
Morphotype 1	0.773	0.548	Phase I	0.801	**0.049**
Morphotype 2		0.803	Phase II		0.173
